# AMPK-induced novel phosphorylation of RUNX1 inhibits STAT3 activation and overcome imatinib resistance in chronic myelogenous leukemia (CML) subjects

**DOI:** 10.1038/s41420-023-01700-x

**Published:** 2023-10-30

**Authors:** Meher Bolisetti Gayatri, Rama Krishna Kancha, Abhayananda Behera, Dorababu Patchva, Nagaraj Velugonda, Sadasivudu Gundeti, Aramati Bindu Madhava Reddy

**Affiliations:** 1 Department of Animal Biology, School of Life Sciences, University of Hyderabad, Hyderabad, 500046 India; 2 Molecular Medicine and Therapeutics Laboratory, CPMB, Osmania University, Hyderabad, 500007 India; 3 Department of Pharmacology, Apollo Institute of Medical Sciences and Research, Jubilee Hills, Hyderabad, 500033 India; 4 Department of Medical Oncology, Nizam’s Institute of Medical Sciences, Hyderabad, 500082 India

**Keywords:** Chronic myeloid leukaemia, Translational research

## Abstract

Imatinib resistance remains an unresolved problem in CML disease. Activation of JAK2/STAT3 pathway and increased expression of RUNX1 have become one reason for development of imatinib resistance in CML subjects. Metformin has gained attention as an antileukemic drug in recent times. However, the molecular mechanism remains elusive. The present study shows that RUNX1 is a novel substrate of AMP-activated kinase (AMPK), where AMPK phosphorylates RUNX1 at Ser 94 position. Activation of AMPK by metformin could lead to increased cytoplasmic retention of RUNX1 due to Ser 94 phosphorylation. RUNX1 Ser 94 phosphorylation resulted in increased interaction with STAT3, which was reflected in reduced transcriptional activity of both RUNX1 and STAT3 due to their cytoplasmic retention. The reduced transcriptional activity of STAT3 and RUNX1 resulted in the down-regulation of their signaling targets involved in proliferation and anti-apoptosis. Our cell proliferation assays using in vitro resistant cell line models and PBMCs isolated from CML clinical patients and normal subjects demonstrate that metformin treatment resulted in reduced growth and improved imatinib sensitivity of resistant subjects.

## Introduction

Runt-related transcription factor 1 (RUNX1) is a major transcription factor in maintaining and lineage commitment of hematopoietic stem cells [1]. Somatic mutations in *RUNX1* are associated with the progression of chronic myelogenous leukemia (CML) to blast crisis [2], and chromosomal translocations resulting in the generation of fusion genes such as *RUNX1-ETO*, *RUNX1-EVII*, etc., are frequently reported in leukemia [3]. Initially, it was thought that fusion proteins act as negative regulators for wild-type RUNX1 in a dominant-negative fashion, which enables CML’s progression to blast crisis phase by the generation of drug resistance [3–6]. However, recent studies have shown that even in the absence of fusion proteins, RUNX1 acts as an oncogene by cooperating with BCR-ABL to induce blast crisis phase like phenotype in CML cases [7]. There is growing evidence suggesting an oncogenic role of wild-type RUNX1 in the case of CML, nevertheless, the underlying molecular mechanisms remain elusive [7, 8]. Imatinib-mesylate is a gold standard drug for CML treatment, where it acts as a competitive inhibitor for BCR-ABL kinase [9]. However, the development of imatinib resistance is increasingly reported in CML patients [10]. Janus kinases (JAK) / signal transducer and activator of transcription proteins (STAT) is one of the significant pathways that drive imatinib resistance along with RUNX1 [11]. Consistent activation of BCR-ABL results in activation of the JAK/STAT pathway through phosphorylation, which in turn activates a cascade of genes involved in proliferation and anti-apoptotic signaling, resulting in the generation of imatinib resistance [12, 13]. RUNX1 promotes STAT3 activation in keratinocytes through repression of Suppressor of cytokine signaling 3 (SOCS3), a negative regulator of STAT3 phosphorylation [14, 15].

Metformin is an anti-glycemic drug that recently emerged as an antileukemic drug owing to its ability in inhibiting mTORC1 (mammalian Target Of Rapamycin Complex 1) through AMPK activation [13, 16]. However, the role of metformin in inhibiting the JAK/STAT pathway and in overcoming imatinib resistance is unexplored. Our recent work on RUNX2 has shown that RUNX proteins serve as possible AMPK substrates [17]. In the light of the above knowledge, we have analyzed the effect of metformin on activation of RUNX1 and JAK/STAT pathway, which are major drivers of imatinib resistance. In the present study, we show that metformin inhibits both RUNX1 and STAT3 signaling through AMPK resulting in improved imatinib sensitivity.

## Results

### RUNX1 is a novel substrate of AMPK

**Fig. 1:**
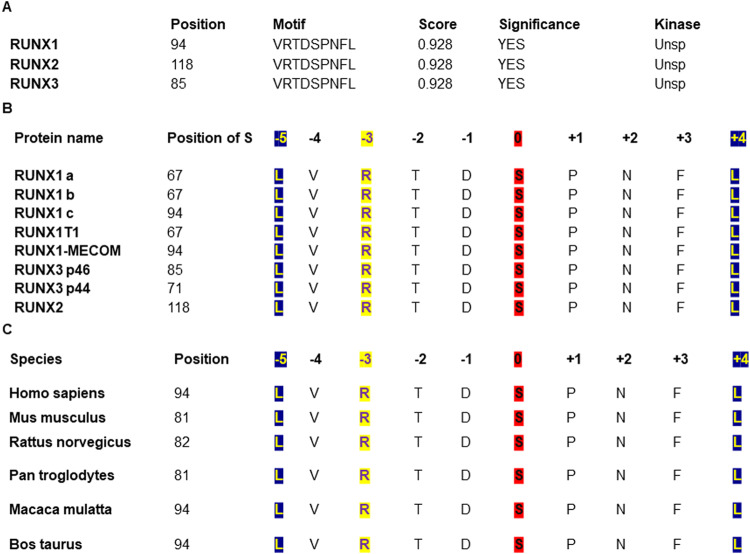
In silico AMPK binding motif prediction in RUNX1. **A** List of putative AMPK binding motif present on RUNX proteins along with the scores as predicted using phospho motif finder (2018). **B** Conservation of putative AMPK binding motif across RUNX family splice variants and fusion proteins. **C** Conservation of putative AMPK binding motif present on RUNX1 across species consolidated using ClustalW2 protein sequence alignment. Unsp unspecified, L lysine, V valine, R arginine, T threonine, D aspartic acid, S serine, P proline, N asparagine, F phenylalanine, A alanine.

In our earlier work, we have reported the presence of AMPK binding motif in the DNA binding runt domain of RUNX2. Since the runt homology domain is conserved across the RUNX family, we have analyzed the presence of AMPK binding motif in RUNX1 and its fusion proteins and RUNX3 using phospho motif finder. RUNX1, 3 (Fig. 1A) and RUNX1 fusion proteins (Fig. 1B) have AMPK binding motif indicating the possibility of them acting as potential physiological AMPK substrates. The AMPK binding motif present on RUNX1 is conserved across species showing the physiological and evolutionary significance of RUNX1 and AMPK interactions (Fig. 1C).

### AMPK phosphorylates RUNX1 at Ser 94 position

**Fig. 2:**
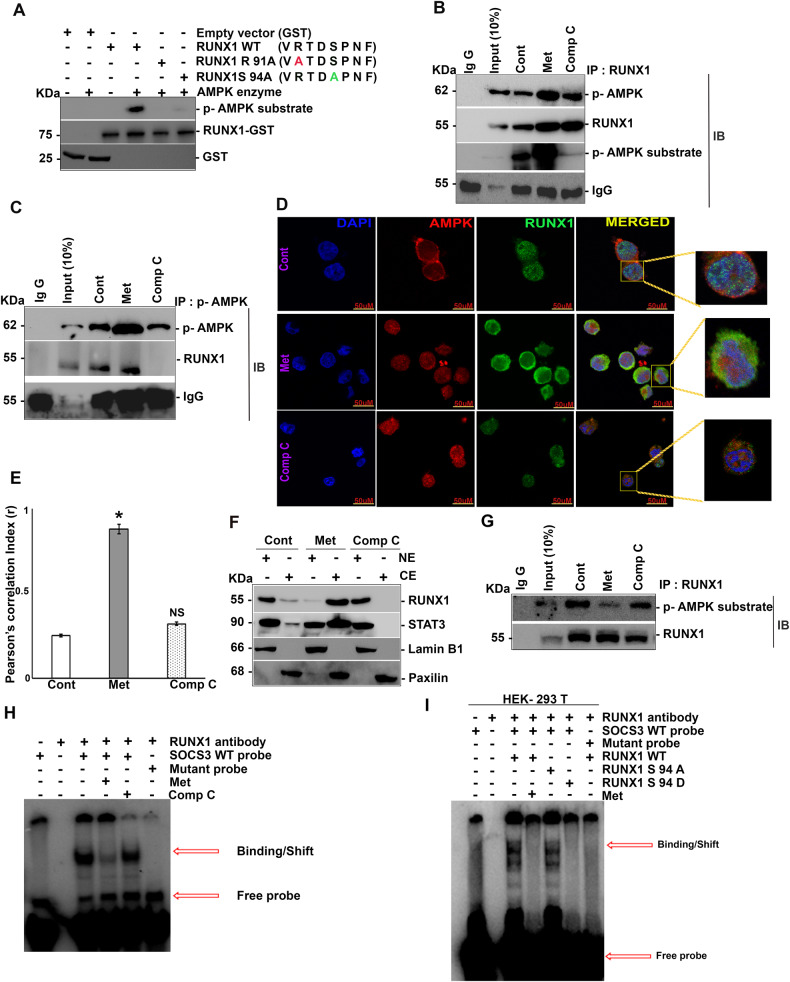
AMPK phosphorylates RUNX1 at Ser 94 position. **A** In vitro phosphorylation of RUNX1 by AMPK at Ser 94 position as detected by p-AMPK substrate-specific antibody. **B** Immunoprecipitation analysis of endogenous RUNX1 with p-AMPK in K562 WT cells following treatment with either metformin (10 mM) or compound C (5 μM) for 6 h, showing the increased physical interaction between RUNX1 and p-AMPK upon metformin treatment. **C** Co-immunoprecipitation analysis of endogenous p-AMPK with RUNX1 in K562 WT cells post to treatment with either metformin (10 mM) or treatment with compound C (5 μM) for 6 h, showing the increased physical interaction between RUNX1 and p-AMPK in metformin-treated cells. **D** Co-localization analysis showing the increased physical interaction between endogenous RUNX1 (Alexa 488) and AMPK (Alexa 546) in the presence of metformin treatment. **E** Quantification of immunofluorescence data using Image J software on three independent fields of separate experiments. **F** Sub-cellular fractionation of K562 WT cells showing decreased nuclear localization of RUNX1 and STAT3 in metformin-treated conditions (following same treatment regime as above). **G** K562 WT cells were subjected to immunoprecipitation analysis of endogenous RUNX1 with p-AMPK substrate-specific antibody post to nuclear fraction isolation, showing decreased phospho-RUNX1 in metformin treated lanes. **H** K562 WT nuclear extracts were isolated post to treatment with either metformin (10 mM) alone or with compound C (5 μM) alone or none for 6 h and subjected to EMSA with SOCS3 probe, resulting in decreased binding of RUNX1 in metformin-treated cells. **I** HEK-293 T cells were transfected with RUNX1 WT, S94A and S94D, with or without metformin (10 mM) treatment post to transfection (after 48 h) for 6 h and nuclear extracts were subjected to EMSA, showcasing decreased binding in phospho-mimetic mutant. *N* = 3, Mean ± SEM *p < 0.05 versus control. Cont Control, Met Metformin, Comp C Compound C, CE Cytoplasmic extract, NE Nuclear extract, IP Immunoprecipitation, IB Immunoblotting, WT Wild type, NS Non-Significant.

In order to validate the AMPK binding motif, present in RUNX1, in vitro AMPK kinase assay was carried out in a cell free system. RUNX1 served as a substrate of AMPK and phosphorylation of Ser 94 was confirmed by site directed mutagenesis (Fig. 2A). As RUNX1 plays a critical role in CML pathogenesis, we have used K562 cells to confirm the phosphorylation of RUNX1 by AMPK. Upon metformin treatment of K562 cells, enrichment of p-AMPK in RUNX1 immunoprecipitates was observed, which was not seen when cells were treated with compound C (an inhibitor of p-AMPK) (Fig. 2B). The phosphorylation of RUNX1 by p-AMPK was confirmed by immunoblotting with AMPK substrate-specific antibody on RUNX1 immunoprecipitated lysates (as p-RUNX antibody was commercially unavailable). AMPK substrate-specific antibody signal was enriched in metformin-treated lanes post to immunoprecipitation by RUNX1 (Fig. 2B), suggesting RUNX1 as a physiological substrate of AMPK. The interaction between RUNX1 and p-AMPK was further validated by immunoprecipitation of p-AMPK, following treatment with either metformin or compound C. Enrichment of RUNX1 in metformin-treated lysates indicating RUNX1 as a substrate of AMPK (Fig. 2C) was observed. This interaction was further confirmed by immunofluorescence analysis of K562 (wild type) cells post to treatments with either metformin or compound C (Fig. 2D, E).

We next analyzed the effect of RUNX1 Ser 94 phosphorylation on nuclear localization and transactivation function of RUNX1. K562 cells treated with either metformin or compound C and subjected to subcellular fractionation. Upon metformin treatment, nuclear localization of RUNX1 was reduced along with increased cytoplasmic retention (Fig. 2F & Supplementary Fig. 1B), which was not seen in compound C treated cells. Similar trend was observed in STAT3 localization (Fig. 2F & Supplementary Fig. 1C). We then analyzed the levels of phosphorylated RUNX1 present in nuclear compartments, for this K562 WT cells were treated with metformin and subjected to immunoprecipitation by RUNX1 in nuclear fraction. Phosphorylated RUNX1 was greatly reduced in metformin treated lanes (Fig. 2G). As AMPK induced phosphorylation was observed on the DNA binding domain of RUNX1, we next analyzed the DNA binding ability of phosphorylated RUNX1. Since RUNX1 was known to promote STAT3 activation through suppression of SOCS3, we have assessed the effect of RUNX1 Ser 94 phosphorylation on SOCS3 expression. Analysis of SOCS3 promoter revealed the presence of RUNX1 binding consensus at −355bp upstream. Thus, we have analyzed RUNX1 binding on SOCS3 promoter in response to metformin treatment. Upon metformin treatment, RUNX1 binding on SOCS3 promoter was lost (Fig. 2H). The role of RUNX1 Ser 94 phosphorylation was further confirmed by transfecting HEK-293T (which were known to have either low or no endogenous RUNX1 levels) cells with RUNX1 wild type, RUNX1 S 94A (phospho-null mutant) and RUNX1 S94D (phospho-mimetic mutant) variants and nuclear extracts were subjected to EMSA. It could be seen that RUNX1 WT and phospho-null mutant had a potent binding to SOCS3 promoter compared to RUNX1 WT treated with metformin or phospho-mimetic mutant (Fig. 2I).

### AMPK inhibits STAT3 activation through RUNX1 Ser 94 phosphorylation

**Fig. 3:**
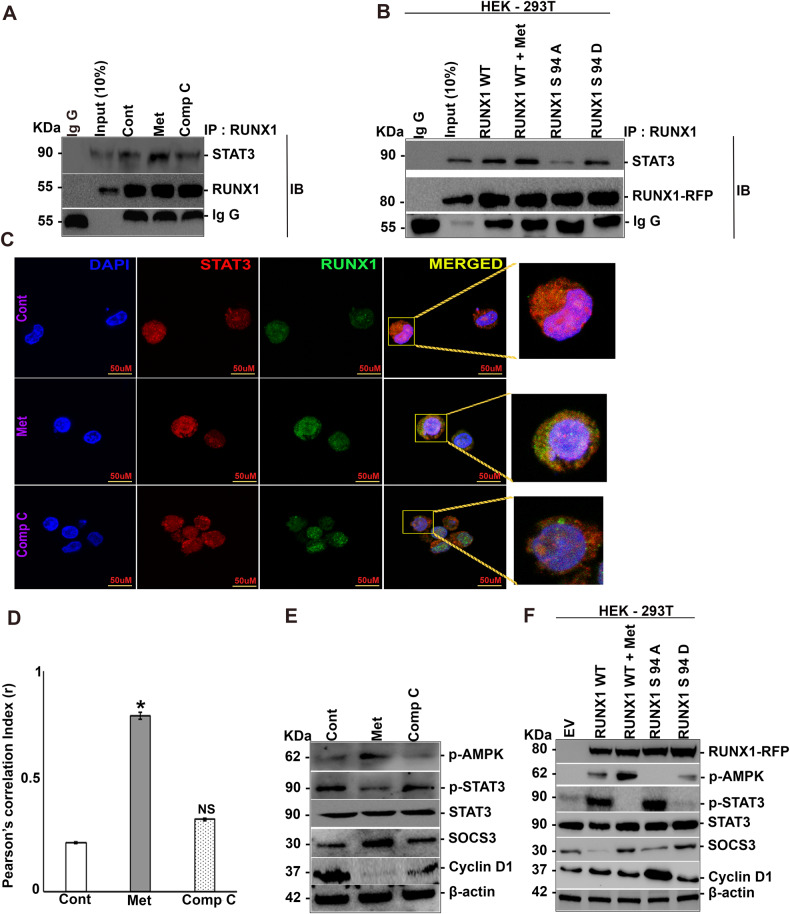
Phospho-RUNX1 inhibits STAT3 phosphorylation in K562 WT cells. **A** Immunoprecipitation analysis of endogenous RUNX1 with STAT3 in K562 WT cells showing the increased physical interaction between RUNX1 and STAT3 following metformin treatment. **B** Immunoprecipitation analysis of ectopically expressed RUNX1 mutants with endogenous STAT3 in HEK-293T cells in the presence of metformin (10 mM, for 6 h, given 48 h after transfection) showed increased physical interaction between RUNX1 WT and STAT3 in metformin-treated WT cells as well as phospho-mimetic mutant cells. **C** Co-localization analysis showing the increased physical interaction between endogenous RUNX1 (Alexa 488) and STAT3 (Alexa 546) in the presence of metformin. **D** Quantification of immunofluorescence data using Image J software on three independent fields and experiments. **E** Immunoblot analysis of K562 WT showing levels of p-STAT3, STAT3, SOCS3, and Cyclin D1 in response to treatment with metformin (10 mM) or compound C (5 μM) for 12 h or none. **F** Immunoblot analysis of HEK-293T cells transfected with either RUNX1 WT or RUNX1 S94A or RUNX1 S94D with or without metformin (10 mM) treatment following transfection (after 48 h) for 12 h showing levels of p-STAT3, STAT3, SOCS3, and Cyclin D1. *N* = 3, Mean ± SEM *p < 0.05 versus control. Cont Control, Met Metformin, Comp C Compound C, EV empty vector, IP Immunoprecipitation, IB Immunoblotting, WT Wild type, NS Non-Significant.

In our earlier section it was observed that metformin treatment resulted in increased cytoplasmic retention of both RUNX1 and STAT3. Thus, we analyzed the interaction between RUNX1 and STAT3 through immunoprecipitation, because one of the reasons behind increased cytoplasmic retention could be physical sequestration of RUNX1 and STAT3. Enrichment of STAT3 in immunoprecipitated lysates of RUNX1 was observed in metformin-treated cells but not in compound C treated cells (Fig. 3A). The effect of RUNX1 Ser 94 phosphorylation on interaction between RUNX1 and STAT3 was further established by transfecting HEK-293T cells with RUNX1 WT, phospho-null and phospho-mimetic variants followed by immunoprecipitation with RUNX1; Enrichment of STAT3 was seen in RUNX1 WT treated with metformin and in phospho-mimetic mutant but was lost in case of phospho-null mutant (Fig. 3B). The interaction between RUNX1 and STAT3 was further studied in K562 (WT) cells by immunofluorescence (Fig. 3C, D). Metformin treatment resulted in reduced nuclear localization of both RUNX1 and STAT3, resulting in reduced expression of their target genes such as SOCS3, Cyclin D1, D3 and BCL2 at both mRNA (SF 1A) and protein level (Fig. 3E; Supplementary Fig. 1D, E). The role of RUNX1 Ser 94 phosphorylation in mediating the inhibitory effects of AMPK was confirmed by transfecting HEK-293T cells with RUNX1 WT, phospho-null and phospho-mimetic variants, followed by immunoblotting for STAT3 targets. Activation of STAT3 signaling (measured through p (Y705) -STAT3) and expression of STAT3 targets was lost in RUNX1 wild type treated with metformin and in phospho-mimetic mutant whereas it was increased in phospho-null mutant (Fig. 3F & Supplementary Fig. 1F). Correlating with the phosphorylation status of STAT3 (Supplementary Fig. 1G), SOCS3 levels were increased in RUNX1 wild type treated with metformin and in phospho-mimic mutant but was decreased in phospho-null mutant (Fig. 3F & Supplementary Fig. 1F).

### RUNX1 serves as an AMPK substrate in imatinib resistance cells

**Fig. 4:**
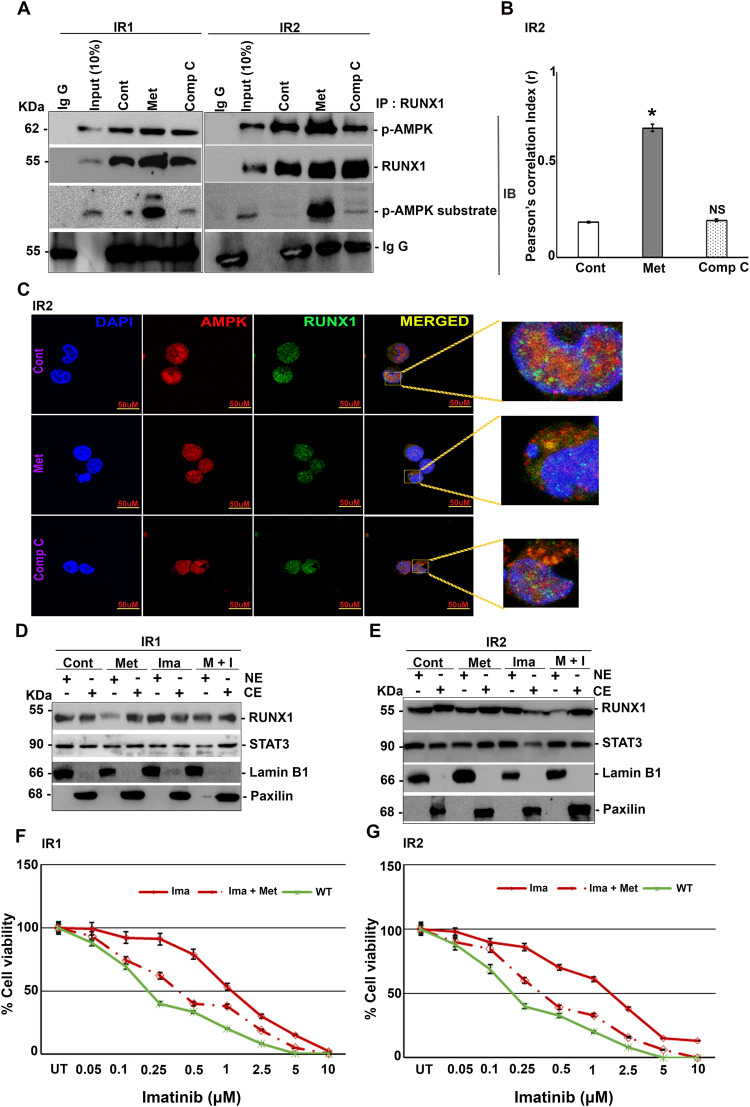
Metformin improves imatinib sensitivity of resistant lines (IR1 and IR2) through the p-AMPK/ RUNX1 axis. **A** Immunoblot analysis of endogenous RUNX1 with p-AMPK in K562 IR1 and IR2 cells post to treatment with either metformin (10 mM) alone or with compound C (5 μM) for 6 h, showing increased physical interaction in the metformin-treated lane. **B** Quantification of immunofluorescence data using Image J software on three independent fields and experiments. **C** Co-localization analysis showing the increased physical interaction between endogenous RUNX1 (Alexa 488) and AMPK (Alexa 546) in the presence of metformin in K562 IR2 cells (treatment regime is same as above). **D** Sub-cellular fractionation of K562 IR1 cells showing with metformin (10 mM) alone or with imatinib (1 μM) or none or both (imatinib 1 μM; metformin 10 mM) for 6 h, showcasing decreased nuclear localization of RUNX1 and STAT3 in metformin-treated conditions. **E** Sub-cellular fractionation of K562 IR2 cells following similar treatment conditions, showing decreased nuclear localization of RUNX1 and STAT3 in metformin-treated conditions. **F** Cell viability analysis of K562 IR1 cells post to treatment with imatinib alone or in combination with metformin (0.25 mM) for 72 h with K562 WT as control showing increased imatinib sensitivity. **G** Cell viability analysis of K562 IR2 cells post to treatment with imatinib alone or in combination with metformin (0.25 mM) for 72 h with K562 WT as control showing increased imatinib sensitivity. *N* = 3, Mean ± SEM *p < 0.05 versus control. Cont Control, Met Metformin, Ima: imatinib, M + I metformin plus imatinib, CE Cytoplasmic extract, NE Nuclear extract, IP Immunoprecipitation, IB Immunoblotting, WT Wild type, NS Non-Significant.

RUNX1 is one of the known drivers involved in imatinib resistance [18]. Therefore, we analyzed if RUNX1 and AMPK interactions are present in imatinib-resistant cell lines derived from clonal evolution. For this, we have used imatinib-resistant K562 cells, namely K562-IR1 and K562- IR2 (hereafter referred to as IR1 and IR2), both having differential sensitivities towards imatinib (IC50: 1.2 ± 0.2 μM and 2.2 ± 0.1 μM respectively). Both IR1 and IR2 were treated with either metformin or compound C and subjected to immunoprecipitation by RUNX1 antibody. Both in IR1 and IR2, there was an enrichment of p-AMPK in metformin-treated lysates, which was not seen in compound C treatment (Fig. 4A). Immunoblotting with p-AMPK substrate-specific antibody revealed that RUNX1 was a substrate of AMPK in both IR1 and IR2 (Fig. 4A). This interaction was further confirmed by immunofluorescence in both IR1 (Supplementary Fig. 2A, B) and IR2 (Fig. 4B, C) cells. We then analyzed if RUNX1 Ser 94 phosphorylation inhibits the nuclear localization of RUNX1 and STAT3 in imatinib-resistant cells. Upon treatment with metformin alone or in combination with imatinib, there was a decrease in nuclear localization of both RUNX1 and STAT3 in IR1 (Fig. 4D; Supplementary Fig. 2C, D) and IR2 (Fig. 4E; Supplementary Fig. 2E, F). However, when treated with imatinib alone, nuclear localization of RUNX1 and STAT3 was high compared to untreated in IR1 (Fig. 4D; Supplementary Fig. 2C, D) and IR2 (Fig. 4E; Supplementary Fig. 2E, F). Both RUNX1 and STAT3 regulate cell proliferation; thus, we next analyzed the effect of metformin treatment on the viability of K562 WT, IR1 and IR2 cells. K562 cells were treated with imatinib alone or in combination with metformin. Compared to WT, both IR1 and IR2 were resistant to imatinib alone treatment whereas in presence of metformin, the sensitivity of both IR1 and IR2 towards imatinib has increased by decreasing the IC50 values of IR1 to 0.75 ± 0.1 μM from 1.2 ± 0.2 μM and for IR2 to 0.7 ± 0.2 μM from 2.2 ± 0.2 μM (Fig. 4F, G).

### RUNX1 Ser 94 phosphorylation inhibits STAT3 activation in imatinib-resistant cells

**Fig. 5:**
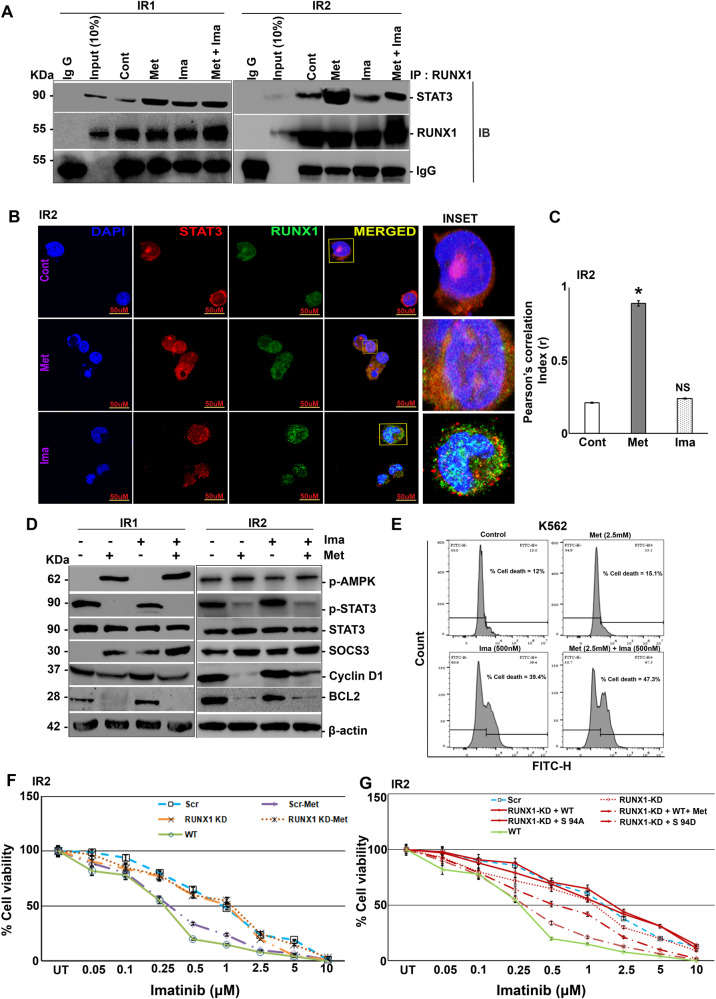
Metformin inhibits STAT3 phosphorylation through RUNX1 Ser 94 phosphorylation. **A** Immunoprecipitation analysis of endogenous RUNX1 with STAT3 in K562 IR1 and IR2 cells post to treatment with either metformin (10 mM) alone or imatinib (1 μM) alone or none or both for 6 h, showing increased physical interaction in the metformin-treated lane. **B** Co-localization analysis in K562 IR2 cells with similar treatment conditions as above for metformin and imatinib, showing the increased physical interaction between endogenous RUNX1 (Alexa 488) and STAT3 (Alexa 546) in the presence of metformin. **C** Quantification of immunofluorescence data using Image J software on three independent fields and experiments. **D** Immunoblot analysis of K562 IR1 & IR2 showing levels of p-STAT3, STAT3, SOCS3, Cyclin D1, and BCL2 in response to metformin (10 mM) treatment alone or along with imatinib (1 μM) or none for 12 h. **E** Flow cytometry analysis for DAD-BODIPY/PI staining to measure the apoptosis rate of K562 cells treated with metformin, imatinib, or their combination. **F** Cell viability analysis of K562 IR2 cells transduced with either Scr or RUNX1 KD with or without metformin (0.25 mM) along with imatinib treatment, using WT as control. **G** Cell viability analysis of K562 IR2-RUNX1-KD, RUNX1-WT, RUNX1-S94A, and RUNX1-S94D transduced cells in response to imatinib treatment alone or along with metformin (0.25 mM) for 72 h with K562 WT as control, showing decreased cell viability in the presence of metformin and in RUNX1-S94D cells which was reversed upon RUNX1-KD and in RUNX1-WT and RUNX1-S94A. *N* = 3, Mean ± SEM *p < 0.05 versus control. Cont Control, Met Metformin, Ima imatinib, Scr scrambled, KD knock down, IP Immunoprecipitation, IB Immunoblotting, WT Wild type, IR Imatinib resistance, NS Non-Significant.

IR1 and IR2 cells were treated with either metformin or imatinib, either alone or in combination, and subjected to immunoprecipitation by RUNX1. STAT3 enrichment was observed in lysates treated with either metformin alone or in combination with imatinib compared to untreated and imatinib alone (Fig. 5A). This was further validated by immunofluorescence in both IR1 (Supplementary Fig. 3A, B) and IR2 (Fig. 5B, C) cells. IR1 and IR2 cells treated with either metformin or imatinib alone or in combination were subjected to immunoblotting. Following metformin treatment, either alone or in combination with imatinib, the activation of STAT3 (Fig. 5D & Supplementary Fig. 3G) and its downstream targets like Cyclin D1 and BCL2 were reduced compared to untreated and imatinib alone (Fig. 5D; Supplementary Fig. 3C–F). However, SOCS3 levels were increased in metformin treatment alone or in combination with imatinib (Fig. 5D & Supplementary Fig. 3C–F). Therefore, we analyzed the effect dose dependant cell growth upon metformin treatment which showed reduced cell growth (Supplementary Fig. 4A) and thus analyzed apoptosis using DAD^(R)^KIT as described in methods, where it showed 47% of apoptotic cells in WT cell lines in combination of Imatinib with metformin compared to 15% in metformin and 39% in imatinib alone (Fig. 5E). Western blot analysis showed activation of Bim, a proapoptotic protein, and reduced levels of pBcl2, an antiapoptotic protein was observed in concordance with the above results in a dose dependent manner in WT, IR1 and IR2 cell lines (Supplementary Fig. 4B). We next analyzed the role of RUNX1 in mediating the anti-proliferative effects of metformin. RUNX1 knockdown was carried out in both IR1 and IR2 cells using shRNA’s mediated RUNX1 knockdown stable cell lines. Out of shRNA 1 and shRNA 2, the efficiency of knockdown by shRNA 2 was significant, thus we have used shRNA 2 stable lines for all further experiments (Supplementary Fig. 5A, B). Upon RUNX1 knockdown, the anti-proliferative effects of metformin were lost (Fig. 5F & Supplementary Fig. 5G). IC50 for imatinib has increased to 1 ± 0.2 μM for IR1from 0.3 ± 0.1 μM (calculated for imatinib treatment along with metformin) post RUNX1 knockdown, which is similar to imatinib alone treated scenario (Supplementary Fig. 5G). Similar results were observed in IR2 where IC50 has increased to 1.25 ± 0.2 μM from 0.75 ± 0.1 μM (Fig. 5F). In both IR1 and IR2 RUNX1 knockdown has resulted in marginal reduction of cell viability, as RUNX1 WT is known to promote cell proliferation in leukemia [19]. To decipher the role of RUNX1 Ser 94 phosphorylation in mediating metformin effects, RUNX1 WT, RUNX1 S 94 A and RUNX1 S 94 D were expressed in RUNX1 knockdown IR1 and IR2 cells (Supplementary Fig. 5C–F), followed by treatment with imatinib alone or in combination with metformin. RUNX1 KD resulted in reduction of imatinib IC50 by a factor of 0.25, which was reverted to original when RUNX1 WT expression was restored in both IR1 (Supplementary Fig. 5H) and IR2 (Fig. 5G). However, when treated with metformin along with imatinib in RUNX1 WT overexpressed cells, imatinib sensitivity has improved as IC50 values changed to 0.3 ± 0.1 μM and 0.75 ± 0.18 μM for IR1 and IR2 respectively. But when cells were transfected with phospho-null variant, there was a decrease in imatinib sensitivity as IC50 values were near to that of control (scrambled) in both IR1 and IR2 cells. The effect was reversed when IR1 and IR2 were transfected with phosphomimic variant, which followed the trend similar to K562 WT (Supplementary Fig. 5H & Fig. 5G). In line with reduced cell proliferation, STAT3 phosphorylation was reduced in both IR1, and IR2 transfected with RUNX1 shRNA-2 and RUNX1 S 94 D, with contaminant increase in SOCS3 expression (Supplementary Fig. 5C–F).

### Metformin improves imatinib sensitivity in imatinib-resistant subjects through AMPK/RUNX1 S 94 axis

**Fig. 6:**
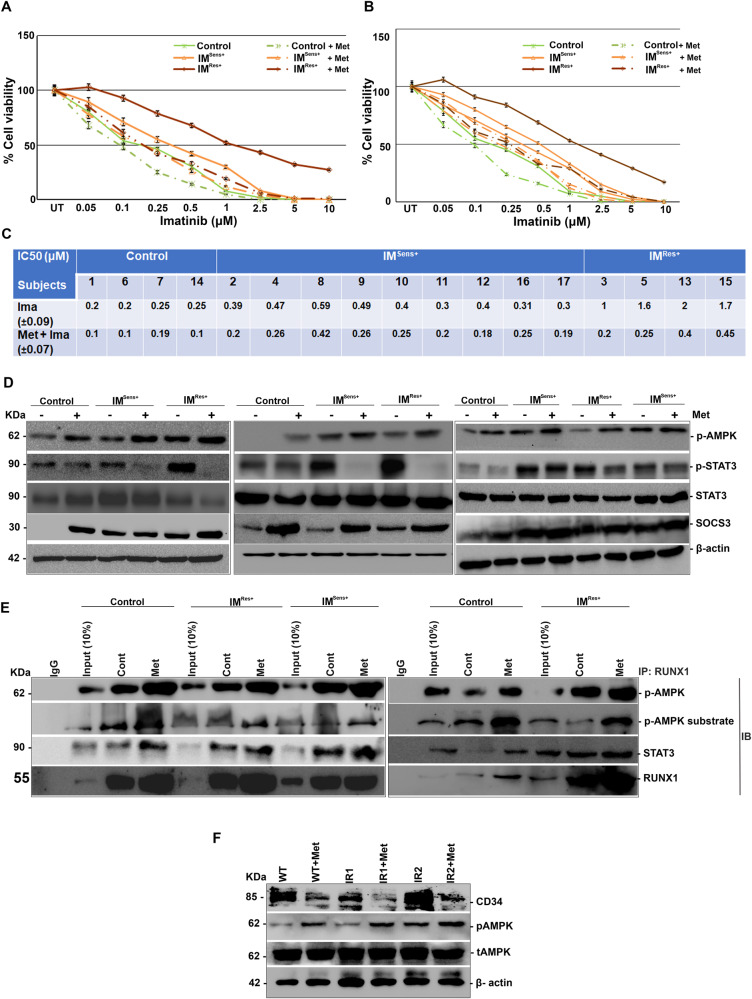
Metformin reduces the viability of PBMCs from CML subjects through AMPK/ RUNX1 Ser 94/ STAT3 axis. **A**, **B** Cell viability analysis of PBMCs post to treatment with imatinib alone or in combination with metformin (0.25 mM) for 72 h showing increased imatinib sensitivity. **C** Tabulated IC50 values of PBMCs for imatinib treatment with and without the presence of metformin. **D** Immunoblot analysis of PBMCs showing levels of p-STAT3, STAT3 and SOCS3 in response to metformin (10 mM) treatment for 12 h. **E** Immunoprecipitation analysis of endogenous RUNX1 with p-AMPK and STAT3 in PBMCs post to treatment with metformin (10 mM) for 6 h, showing increased physical interaction in metformin-treated lanes. *N* = 3, Mean ± SEM. **F** Effect of Metformin on CD34 expression cells from WT and IR cells in correlation with AMPK activation. Cont Control, Met metformin, Control healthy subjects, IM ^Sen+^ Imatinib-sensitive subjects, IM ^Res+^ Imatinib-resistant subjects, IP Immunoprecipitation, IB Immunoblotting.

From the above observations, it was clear that metformin could increase imatinib sensitivity through the p-AMPK/ RUNX1 axis by inhibiting activation of the STAT3 pathway. Thus, here we intend to validate the same observation using peripheral blood monocytes (PBMCs) derived from CML subjects and age-matched healthy subjects of sample size *n* = 17, where healthy subjects (controls) (*n* = 4: Subj 1, 6, 7 &14), imatinib-sensitive subjects (IM ^Sen+^) (*n* = 9: Subj 2, 4, 8–12, 16, 17) and imatinib-resistant subjects (IM ^Res+^) (*n* = 4: subj 3, 5, 13 &15) were treated with either imatinib alone or in combination with metformin and cell viability was assessed. Metformin treatment resulted in increased imatinib sensitivity of both imatinib-responsive subjects and imatinib resistant subjects as well as in control subjects (Fig. 6A–C; Supplementary Fig. 6A, B). However, the fold by which imatinib sensitivity was increased is higher in imatinib resistant (IM ^Res+^) subjects where the IC50 was decreased by 4–6-fold upon metformin treatment. IC50 for imatinib in control and imatinib sensitive subjects (IM ^Sen+^) was decreased by 1.25–2 folds in both groups, which is lower when compared to that of imatinib resistant subjects (Fig. 6C). Since, metformin improved imatinib sensitivity we then analyzed the activation of STAT3 pathway. There was a decrease in STAT3 activation in PBMCs derived from both imatinib sensitive and imatinib-resistant subjects post to metformin (10 mM for 12 h) treatment (Fig. 6D; Supplementary Figs. 6C and 7A–D). Simultaneously, there was an increase in SOCS3 expression (Fig. 6D; Supplementary Fig. 7E, F). Next, RUNX1/AMPK and STAT3 interactions were analyzed. Upon treatment with metformin, it was seen that there was an enrichment of p-AMPK and RUNX1 phosphorylation as assessed by p-AMPK substrate-specific antibody in RUNX1-antibody pull-down lysates and along with STAT3 enrichment (Fig. 6E). Since CD34 considered as CML initiating cells [20], we then analyzed the expression levels by western blot, the results showed that the expression of CD34 levels were significantly reduced upon metformin treatment which demonstrates that metformin has potential to kill even CML initiating cells (Fig. 6F).

## Discussion

Our present work demonstrates that RUNX1 is a novel substrate of AMPK. AMPK phosphorylates RUNX1 at Ser 94 position, resulting in decreased nuclear localization of RUNX1. The increased cytoplasmic retention of RUNX1 decreases the transactivation function of RUNX1 and, at the same time increases RUNX1 binding with STAT3. RUNX1 negatively regulates SOCS3 [14, 15], which is a repressor of STAT3 signaling [21] and this repression was lost owing to cytosolic retention of RUNX1 due to metformin treatment which was reversed when treated with compound C. Metformin treatment resulted in cytoplasmic retention of both RUNX1 and STAT3, resulting in downregulation of their target genes. The downstream targets of RUNX1 and STAT3 include proteins that are critical for cell proliferation such as Cyclin D’s [22, 23] and anti-apoptotic regulation like BCL2, MCL1 etc., [24, 25]. Owing to the vast target repertoire of RUNX1 and STAT3, both of them were reported to be critical for CML progression towards BC phase and development of imatinib resistance [26, 27], presenting physiologically challenging scenarios for CML treatment [2, 28, 29]. STAT3 also contributes to the development of imatinib resistance through upregulation of MDR1 expression [30, 31], which is a key contributor for the development of imatinib resistance [32] along with aiding in metabolic alterations [26].

**Fig. 7:**
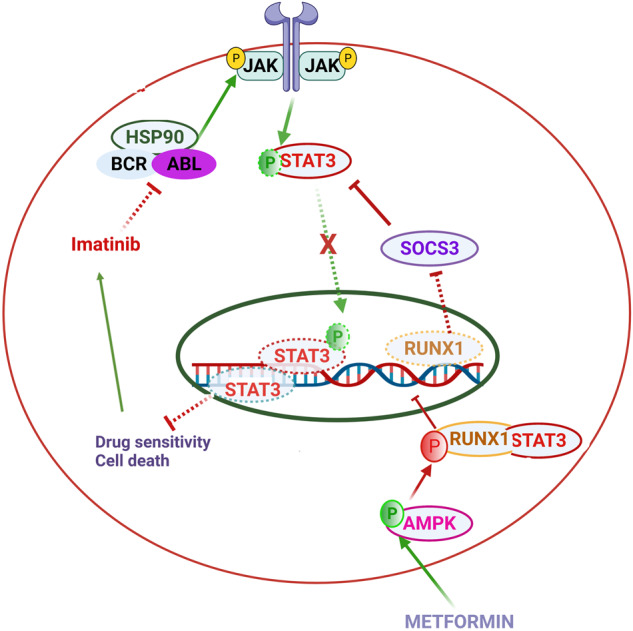
Schematic representation of metformin’s effect on imatinib sensitivity through inhibition of STAT3 activation. The stability of BCR-ABL is maintained by HSP90. Constituent activation of JAK, despite absence of ligand is maintained by BCR-ABL kinase. Active JAK results in activation of STAT3 through tyrosine phosphorylation, which primes STAT3 for dimerization and transcriptional activation, which promotes cell proliferation by inhibiting cell death and decreasing imatinib sensitivity. RUNX1 binds to SOCS3 promoter resulting in transcriptional repression of SOCS3. SOCS3 functions as a negative regulator of STAT3 by promoting STAT3 dephosphorylation. Imatinib inhibits BCR-ABL kinase activity, promoting cell death. Metformin mediated activation of AMPK resulting in RUNX1 phosphorylation at Ser 94 position, which results in increased binding with STAT3. Cytoplasmic retention of STAT3 and phospho-RUNX1 improves imatinib sensitivity and promotes cell death by inhibition of STAT3 activation and transcription of SOCS3.

Our current work shows that AMPK induced phosphorylation of RUNX1 results in a change in RUNX1 interactome and inhibition of transactivation function of RUNX1, which otherwise would cooperate with BCR-ABL conferring growth advantage [7]. The RUNX1 phospho-mimetic and phospho-null mutants have further established the effect of RUNX1 Ser 94 phosphorylation on the growth of imatinib-resistant lines in response to imatinib treatment, where the phospho-null clones had a growth advantage compared to both wild type and phospho-mimetic expressing lines. Metformin treatment also resulted in cytoplasmic retention of STAT3, which is a key player involved in imatinib resistance. Metformin through AMPK/ RUNX1 Ser 94 phosphorylation upregulated SOCS3, which is otherwise repressed by RUNX1 (Fig. 7). SOCS3 results in dephosphorylation of STAT3 [33], which is key to the activation of the STAT3 pathway. The possible role of metformin as an antileukemic drug has been further established using PBMCs derived from CML subjects that were either sensitive or resistant towards imatinib treatment. It was seen that RUNX1 indeed served as an AMPK substrate, and the STAT3 pathway, which was active in imatinib-resistant subjects, was inhibited owing to increased SOCS3 expression, post to metformin treatment. Also, metformin treatment resulted in improved imatinib sensitivity of resistant subjects and reduced levels of CD34 expression. CD34 data suggests that metformin showed anti-proliferative effects on CD34 positive cells which are considered to be tumor initiating cells, however further experiments need to be warranted [20]. Taken together, our data demonstrates the antileukemic effects of metformin and its possible use in the case of imatinib resistant subjects. Our study also establishes RUNX1 as a novel substrate of AMPK. However, it would be of interest to study the effect of AMPK on RUNX1 fusion proteins like AML1T1, AML1EVI1, etc., which also carry the same AMPK binding motif.

## Methods

### In silico analysis

AMPK phosphorylation motif was predicted using PhosphoMotif Finder tool and the efficiency was measured using NetPhos 3.1 tool. The RUNX protein sequence alignment across species and with RUNX family was carried out using ClustalW2 sequence alignment. All sequences were taken from national center for biotechnology information (NCBI) human database and motif positioning corresponds to RUNX1c (NM_001754.5).

### Cell culture and chemicals

Wild type (WT) and imatinib-resistant (IR1 and IR2) K562 cells were maintained in RPMI-1640 (Gibco, USA) medium supplemented with 10% FBS (Gibco, USA) and 1% pen-strep (Gibco, USA) and were grown as mentioned earlier [34]. IR1 and IR2 cells were shown to resistance for imatinib as described earlier [34, 35]. HEK-293 T cells were cultured in DMEM (Gibco, USA) medium supplemented with 10% FBS (Gibco, USA) and 1% pen-strep (Gibco, USA). Puromycin, imatinib, metformin and dorsomorphin (compound C) were procured from Sigma (USA). Imatinib and dorsomorphin were dissolved in DMSO (Finisar, India) whereas metformin and puromycin stocks were made in PBS. shRNA’s used against RUNX1 were shRNA 1 (TRCN0000338427), shRNA 2 (TRCN0000338492) (Sigma, USA).

### Construction of plasmids

RUNX1 cDNA (NM_001754.4) was purchased from DNASU (USA) (HS CD00630733). RUNX1 WT, R91A and S94A mutant constructs were sub-cloned into GST-tagged vector pGEX-4T1 for bacterial protein expression and RUNX1 WT, S94A and S94D constructs were generated in pDsRed1-N1 vector containing red florescent protein for mammalian expression. The list of primers used for sub-cloning into pGEX-4T1 vector were RUNX1 WT: FP-5′ GGAAGATCTATGGCTTCAGACACAGCATA 3′, RP-5′ GAAGTTGGGGTCGTCGGTGCG 3′; RUNX1 R91A: FP-5′ GAGCTGGTGGCCACCGACAGC 3′, RP-5′ GCTGTCGGTGGCCACCAGCTC 3′; RUNX1 S94A: FP-5′ CGCACCGACGCCCCCAACTTC 3′, RP-5′ GAAGTTGGGGGCGTCGGTGCG 3′; and primers used for sub-cloning into pDsRed1-N1 were, RUNX1 WT: FP-5′ CCGCTCGAGATGGCTTCAGAC 3′, RP-5′ CCGCTCGAGTCAGTAGGGCCTCCACAC 3′; RUNX1 S94D: FP-5′ CGCACCGACGACCCCAACTTC 3′, RP-5′ GGAATTCATTCAGTAGGGCCTCCA 3′; RUNX1 S94D: FP-5′ CGCACCGACGACCCCAACTTC 3′, RP-5′ GAAGTTGGGGTCGTCGGTGCG 3′.

### Bacterial protein expression and purification

GST-tagged fusion proteins RUNX1-WT, R91A and S94A were purified using columns packed with GST beads following the protocol described previously [17].

### In vitro AMPK kinase assay

Reaction mixture was prepared containing 5 μg of RUNX1-WT or RUNX1-R 91 A or RUNX1-S94A along with kinase buffer (20 mM HEPES (pH 7.4), 100 μM ATP and 5 μM AMPK, 1 mM dithiothreitol, 10 mM magnesium acetate) and 100 ng of purified AMPK enzyme (Cat #14-840 – EMD Millipore, USA), which was incubated for 30 min at 37 °C. After incubation the complex was subjected to SDS-PAGE analysis followed by immunoblotting.

### Plasmid transfection

5 × 10^4^ cells were seeded per well in a six well plate and were grown to a confluency of 50%. 2 μg of purified plasmid (RUNX1 WT or S94A or S94D) were diluted in 500 μL of OPTI-MEM medium and incubated for 5 min. Concomitantly 1.5 μL of Lipofectamine-3000 (Thermo Fischer, USA) was diluted in 500 μL of OPTI-MEM medium and incubated for 5 min. The diluted plasmid mixture was added to lipofectamine and incubated for 20 min and then added to cells. The cells were grown in OPTI-MEM for six hours, post to which the medium was replaced with complete medium and grown for 48 h.

### Nuclear and cytosolic extraction

Cells were treated with indicated drugs for 6 h, and collected in 1X PBS. Following which they were resuspended in buffer A (20 mM Tris-HCl, pH-7.4, 10 mM NaCl, 3 mM MgCl2) (Sigma, USA) for 15 min on ice and 10% NP-40 was added with vertexing at high speed, then subjected to centrifugation for 10 min at 8000 rpm at 4 °C. The supernatant represents cytosolic fraction. To the pellet buffer B (10 mM Tris- HCl, pH-7.4, 2 mM Na3VO4,0.5 mM DTT, 100 mM NaCl, 1% Triton- X-100, 1 mM EDTA, 5% glycerol, 0.1% SDS, 1 mM NaF, 1 mM EGTA, 0.5% deoxycholate) (Sigma, USA) was added and incubated on ice for 30 min with vertexing at 5 min interval, then subjected to centrifugation at 14000 rpm for 20 min. The supernatant represents nuclear fraction.

### Electrophoretic mobility shift assay (EMSA)

K562-WT cells were treated with indicated drugs and nuclear extracts were prepared as mentioned earlier. The RUNX1 binding consensus 5′ TAGAGACGAGGTTTCACC 3′ on SOCS3 promoter is 2 kb upstream was used as wild type probe and mutant probe 5′ TAGAGACCAAAATTCACC 3′ were labeled by γ-^32^P. The protocol employed was discussed in detail in our earlier work [17].

### shRNA transduction and generation of stable cell lines

shRNA’s for RUNX1 along with Scr shRNA were transfected into HEK-293T cells using Lipofectamine 3000 (Thermo Fischer, USA) as described above. 48 h post-transfection the medium containing lentiviral particles were collected and stored in −80 °C. K562 cells were plated in a 96 well plate and treated with puromycin to determine final concentration of puromycin to be used for selection. Lentiviral particles were thawed on ice, added to K562 cells and centrifuged at 800 × *g* for 30 min at room temperature in medium containing polybrene (8 μg/mL) (Sigma, USA). The pellet was resuspended in fresh medium without polybrene and seeded in a six well plate. 72 h post, the medium was replaced with medium containing puromycin and maintained until death was observed in control cells.

### Flow cytometry analysis and DAD-FITC/PI staining

To identify and quantify the percentage of apoptotic cells, we used the DAD (Dynamic Apoptosis detection (DAD)) (Cat#UR30120, UR Advanced therapeutics-India, www.uratpx.com) kit and analyzed by flow cytometry as described by manufacturer’s instructions. The WT K562 cells treated with different concentrations of treatments of imatinib and metformin were incubated with 5 μl DAD reagent in 800 μl PBS for 30 min along with 1 mg/ml PI solution at room temperature in the dark. Then, we analyzed the cells using flow cytometer (BD LSRFortesa, USA).

### PBMC isolation from CML subjects

Samples from human subjects were collected with prior approval from the Institutional Ethics Committee of Nizam’s Institute of Medical Sciences, Hyderabad and informed patient consent was obtained by adhering to approved protocols (EC/NIMS/2350/2019). 10 mL of blood was collected in heparinized vacutainers (BD, USA) from CML subjects, along with duly signed consent form. PBMCs were isolated using histopaque (Sigma, USA). Patients who show molecular response to 400 mg/day are classified as IR^SEN^ and with no molecular response even at 800 mg/day are considered as IR^RES^. All the patients were in the chronic phase of CML. 10 mL of his opaque was added to a 50 ml tube an hour before the initiation of isolation by slowly adding equal volumes of blood without disturbing the histopaque layer and centrifuged at 400 × *g* for 30 min with zero break in a swing bucket rotor centrifuge. After centrifugation, the upper layer was discarded, interphase was collected separately and washed twice with 1X PBS. The pellet was resuspended to make a single cell suspension and grown in RPMI-1640 medium supplemented with 10% FBS, 2mM L-glutamine (HiMedia, India), 1% non-essential amino acids (Gibco, USA) 1% sodium pyruvate (Gibco, USA) and 1% pen-strep (Gibco, USA).

### Cell viability assay

Cells were seeded at a density of 4 × 10^3^ cells per well in a 96 well plate. Corresponding drug stocks were made and added to RPMI medium to make a final volume of 250 μL and were grown for 3 days, after which 1X alamarBlue (Invitrogen, USA) was added and incubated for 4 h. Post to incubation fluorescence was measured (excitation at 560 nM and emission at 590 nM).

### RNA isolation and real-time PCR (RT-PCR)

Cells were collected by centrifuging at 3 ×10^3 ^rpm for 5 min and washed twice with ice-cold 1X PBS following addition of TRIzol (Thermo Fisher Scientific, USA) for total RNA isolation. Equal amounts of RNA (1 µg) were taken and subjected to cDNA synthesis using iSCRIPT Bio-Rad cDNA synthesis kit (Bio-Rad, USA). Bio-Rad SYBR Green QRT- PCR Master mix (Bio-Rad, USA) was used for real time PCR. The sequence of primers used were CYCLIN D1: FP-5′ AGGTCTGCGAGGGAACAGAAGTG 3′, RP-5′ TGCAGGCGGCTCTTTTTC 3′; CYCLIN D3: FP-5′ CTGGCCATGAACTACCTGGA 3′, RP-5′ CCAGGAAATCATGTGCAATC 3′; SOCS3: FP-5′ GGAGACTTCGATTCGGGACC 3′, RP-5′ GAAACTTGCTGTGGGTGACC 3′; BCL2: FP-5′ ATGTGTGTGGAGAGCGTCAACC 3′, RP-5′ TGAGCAGAGTCTTCAGAGACAGCC 3′ and ACTIN: FP-5′ GAGAGGGAAATCGTGCGTGAC 3′, RP-5′ CATCTGCTGGAAGGTGGACA 3′. The quantification of real time data was done using 2^-Δ ΔCT^ method [35].

### Immunoprecipitation (IP) and Immunoblotting

Cells were collected by centrifuging at 3000 rpm for 5 min and washed twice with ice-cold 1X PBS following addition of either IP lysis buffer (25 mM Tris (pH-7.4), 150 mM NaCl, 1% NP-40, 1 mM EDTA, 5% Glycerol, 1 mM PMSF and leupeptin along with phosphatase inhibitor) or 1X RIPA (with protease and phosphatase inhibitor cocktails (Sigma, USA)) and subjected to protein isolation by centrifugation at 14,000 rpm for 30 min at 4 °C. For pull-down assay, 750 μg (for cell lines) or 500 μg (in PBMCs) of protein was taken along with 1 μg of antibody (p-AMPK, STAT3 or RUNX1) and left at 4 °C overnight for binding. Protein agarose-A-G plus beads (Santa Cruz Biotechnology Inc., USA) were used for precipitating antigen-antibody complexes. Prior to subjecting for binding, beads were equilibrated using 1X TBST (Triton-X-100, 1 M Tris-base pH-7.4, 1 M NaCl). Post to pull down, the lysates were subjected to SDS-PAGE followed by western blotting. Secondary antibodies used were TrueBlot antibodies conjugated to HRP (Rockland Immunochemicals, USA).

For immunoblotting, equal amounts of protein (60 μg) were taken and subjected to SDS-PAGE. RUNX1/AML1, AMPK, Cyclin D1, GST, SOCS3 and β-actin antibodies were procured from Santa Cruz Biotechnology Inc., USA. p-STAT3 (Y705), STAT3 and BCL2 were purchased from Abcam, USA. Lamin B1, paxillin, CD34, p-AMPK and p-AMPK substrate motif antibodies were procured from Cell Signaling Technologies, USA. Corresponding horseradish peroxidase enzyme (HRP) (linked secondary antibodies were from GeNeI labs, India. The signal was detected by Clarity Western ECL blotting substrates (Bio-Rad, USA) and images were processed using Bio-Rad Chemidoc MP system. All the immunoprecipitation followed by immunoblotting experiments are performed twice for experimental confirmation.

### Immunofluorescence

Cells were grown to confluence (80%) on coverslips coated with poly-lysine (Sigma, USA), followed by treatment with either metformin or compound C for indicated time points, and were washed twice with 1X PBS prior to fixation in 4% formalin for 10 min at room temperature. Cells were washed twice in 1X PBS. Prior to blocking, cells were permeabilized in 0.2% TRITON-X-100 (Sigma, USA) for 15 min followed by washes with 1X PBS. Bocking was done in 5% bovine serum (Hi-media, India) for 1 h at room temperature. Cells were stained with RUNX1/AML1 and AMPK or STAT3 for 2 h at room temperature, followed by the respective fluorescence-tagged secondary antibody staining for 1 h (Alexa Fluor 488 and 594, Invitrogen, USA). Cells were counterstained with 4′,6-diamino-2-phenylindole (DAPI) (Thermo Scientific, USA) for nuclei and images were captured using a laser scanning confocal microscope (LSM 780, Carl Zeiss, Germany).

### Statistical analysis

All data points are represented as mean ± SEM. Statistical analysis was done with one-way ANOVA by comparing the difference between mean values. *P* values less than 0.05 were considered to be statistically significant. All data points were done in triplicates and carried out a minimum of set of three independent experiments for all in-vitro studies and patient data except for immunoprecipitations experiments, where it was done in duplicates.

### Supplementary information

Reproducibility Check list

Supplementary Figures

Original Data File

## Data Availability

All the data is available in main text or in supplementary material as stated in the text.
