# Supplementary material for: AMPK-induced novel phosphorylation of RUNX1 inhibits STAT3 activation and overcome imatinib resistance in chronic myelogenous leukemia (CML) subjects
Source: Cell Death Discov. 2023 Oct 30;9:401. doi: 10.1038/s41420-023-01700-x (PMC10616083; doi:10.1038/s41420-023-01700-x)
Supplement: Supplementary file 2 — Supplementary Figures [file 41420_2023_1700_MOESM2_ESM.pdf]

Supplementary figures:

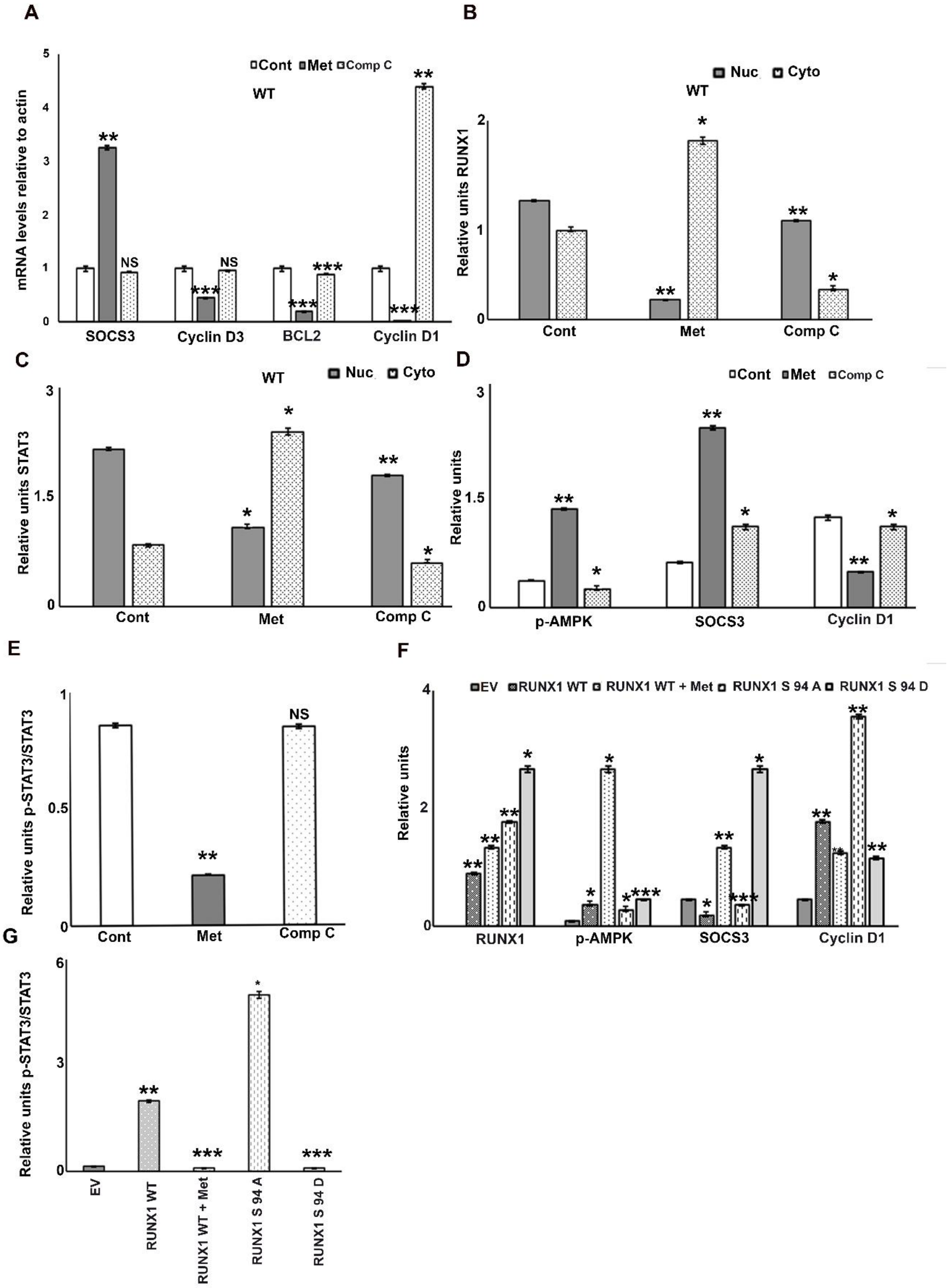

Supplementary figure 1

Supplementary fig. 1: A) RT-PCR analysis of K562 WT cells showing levels of SOCS3, BCL2, Cyclin D1 and D3 in response to metformin (10mM) treatment alone or with compound C (5µM) for 6 hours. **B)** Quantification of RUNX1 immunoblot data of WT cells post metformin and compound C treatment where nuclear values are normalized against Lamin B1 and cytoplasmic values against paxillin. **C)** Quantification of STAT3 immunoblot data of WT cells post metformin and compound C treatment. **D)** Quantification of immunoblot data of WT cells post metformin and compound C treatment for 12 hours showing levels of p-AMPK, RUNX1, SOCS3 and Cyclin D1 normalized to actin. **E)** Quantification of p-STAT3 western blot data of WT cells post metformin and compound C treatment for 12 hours normalized to STAT3. **F)** Quantification of immunoblot data of HEK-293T cells transfected with either RUNX1 WT or RUNX1 S 94 A or RUNX1 S 94 D with or without metformin (10mM) treatment following transfection (after 48 hours) for 12 hours showing levels of p-AMPK, RUNX1, SOCS3 and Cyclin D1 normalized to actin. **G)** and p-STAT3 levels normalized to STAT3. N=3, Mean ± SEM *$p<0.05$ versus control, **$p<0.005$ versus control, ***$p<0.0005$ versus control, $^{NS}p>0.05$ versus control.

Cont: Control, Met: Metformin, Comp C: Compound C, EV: empty vector, WT: wild type, Nuc: nuclear extract, Cyto: Cytoplasmic extract, NS: non-significant.

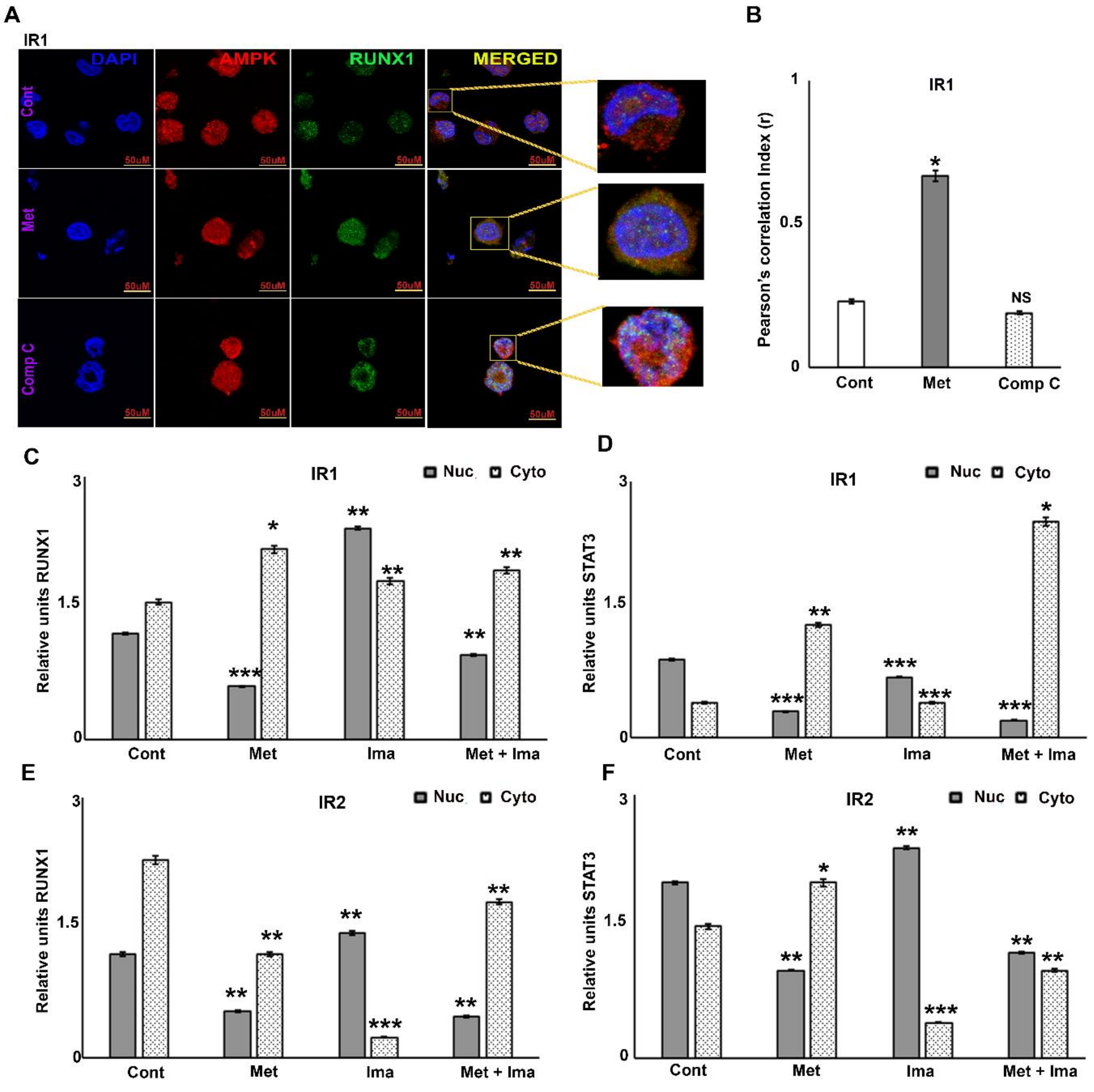

Supplementary figure 2

Supplementary fig. 2: A) Co-localization analysis showing increased physical interaction between endogenous RUNX1 (Alexa 488) and AMPK (Alexa 546) in the presence of metformin (10mM for 6 hours) than compound C (5µM for 6 hours) in K562 IR1 cells. **B)** Quantification of immunofluorescence data in K562 IR1 cells, using Image J software on three independent fields and experiments. K562 IR1 cells treated with metformin (10mM) alone or with imatinib (1µM) or none or both (imatinib 1µM; metformin 10mM) for 6 hours and subjected to quantification of

immunoblot data for **C)** RUNX1 and **D)** STAT3. K562 IR2 cells treated with metformin (10mM) alone or with imatinib (1μM) or none or both (imatinib 1μM; metformin 10mM) for 6 hours and subjected to quantification of immunoblot data for **E)** RUNX1 and **F)** STAT3. N=3, Mean ± SEM *$p<0.05$ versus control, **$p<0.005$ versus control, ***$p<0.0005$ versus control, $^{NS}p>0.05$ versus control.

Cont: Control, Met: Metformin, Comp C: Compound C, Ima: imatinib, Nuc: nuclear extract, Cyto: Cytoplasmic extract, NS: non-significant.

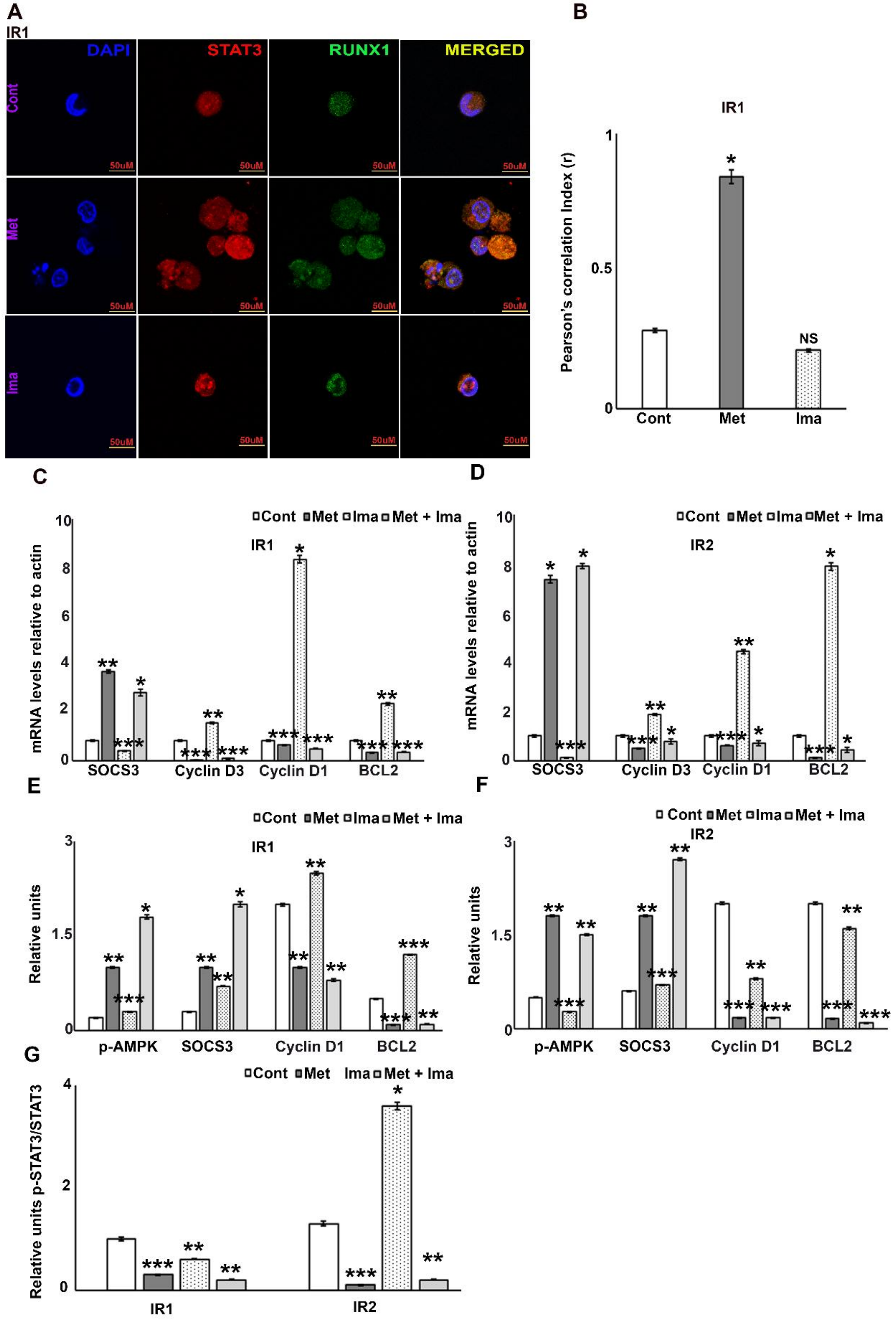

Supplemetary figure 3

Supplementary fig. 3: A) Co-localization analysis showing increased physical interaction between endogenous RUNX1 (Alexa 488) and STAT3 (Alexa 546) in the presence of metformin (10mM for 6 hours) than compound C (5μM for 6 hours) in K562 IR1 cells. **B)** Quantification of immunofluorescence data in K562 IR1 cells, using Image J software on three independent fields and experiments. RT-PCR analysis of **C)** K562 IR1 and **D)** K562 IR2 cells showing levels of SOCS3, BCL-2, Cyclin D1 and D3 in response to metformin (10mM) and or imatinib (1μM) alone or both or none for 6 hours. **E)** K562 IR1 cells treated with metformin (10mM) alone or with imatinib (1μM) or none or both (imatinib 1μM; metformin 10mM) for 12 hours and subjected to quantification of immunoblot data for SOCS3, BCL-2, Cyclin D1 and p-AMPK. **F)** K562 IR2 cells treated with metformin (10mM) alone or with imatinib (1μM) or none or both (imatinib 1μM; metformin 10mM) for 12 hours and subjected to quantification of immunoblot data for SOCS3, BCL-2, Cyclin D1 and p-AMPK. **G)** K562 IR1 and IR2 cells treated with metformin (10mM) alone or with imatinib (1μM) or none or both (imatinib 1μM; metformin 10mM) for 12 hours and subjected to quantification of immunoblot data for p-STAT3.

Mean ± SEM *$p<0.05$ versus control, **$p<0.005$ versus control, ***$p<0.0005$ versus control, $^{NS}p>0.05$ versus control.

Cont: Control, Met: Metformin, Ima: imatinib, NS: non-significant.

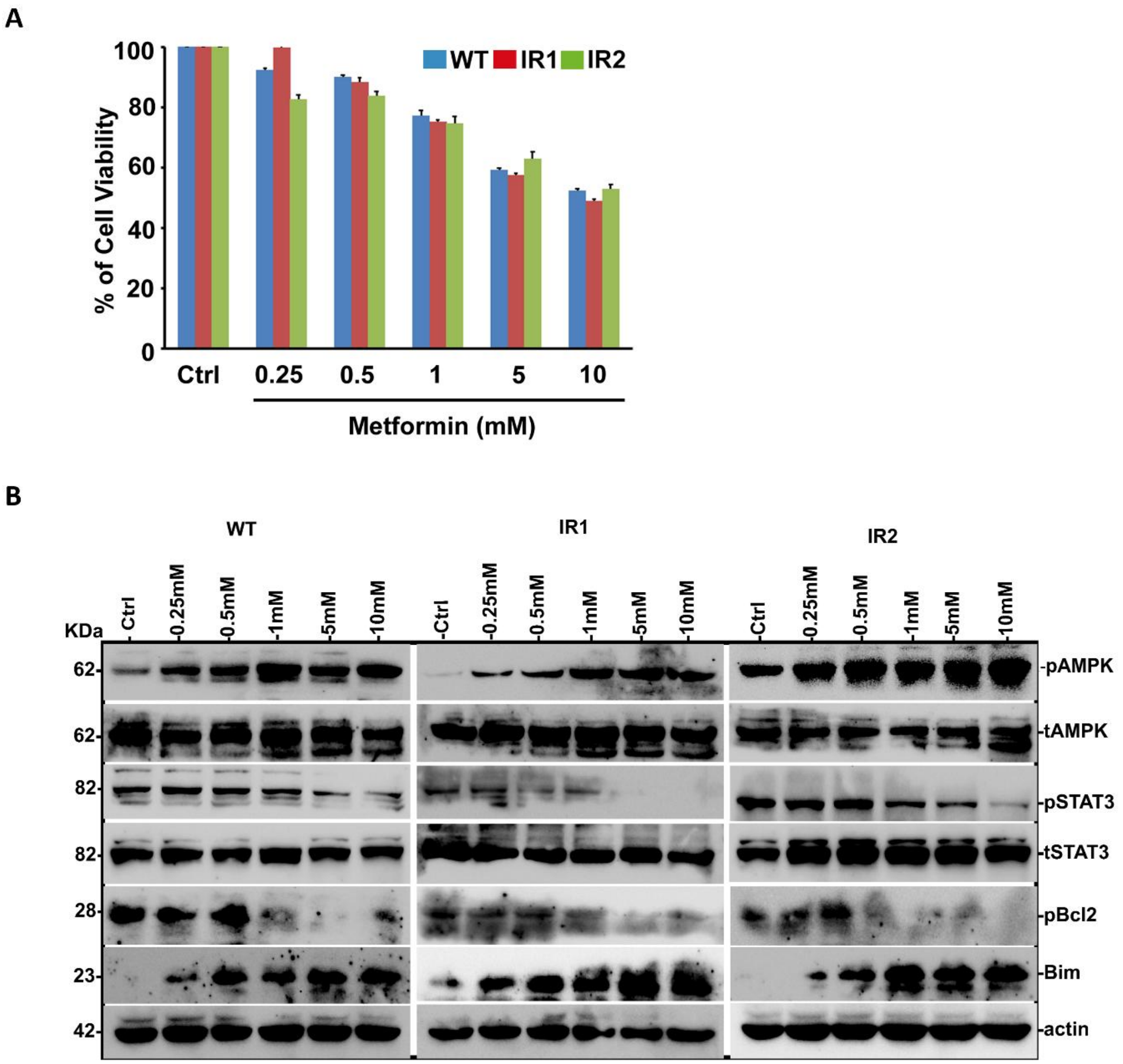

Supplementary figure 4

Supplementary fig. 4: A) Cell proliferation decreases in a dose-dependent manner with metformin treatment in WT, IR1, and IR2 cell lines. **B)** Metformin induces apoptosis by activating pAMPK and reducing pSTAT3 expression in a dose-dependent manner in WT, IR1, and IR2 cell lines.

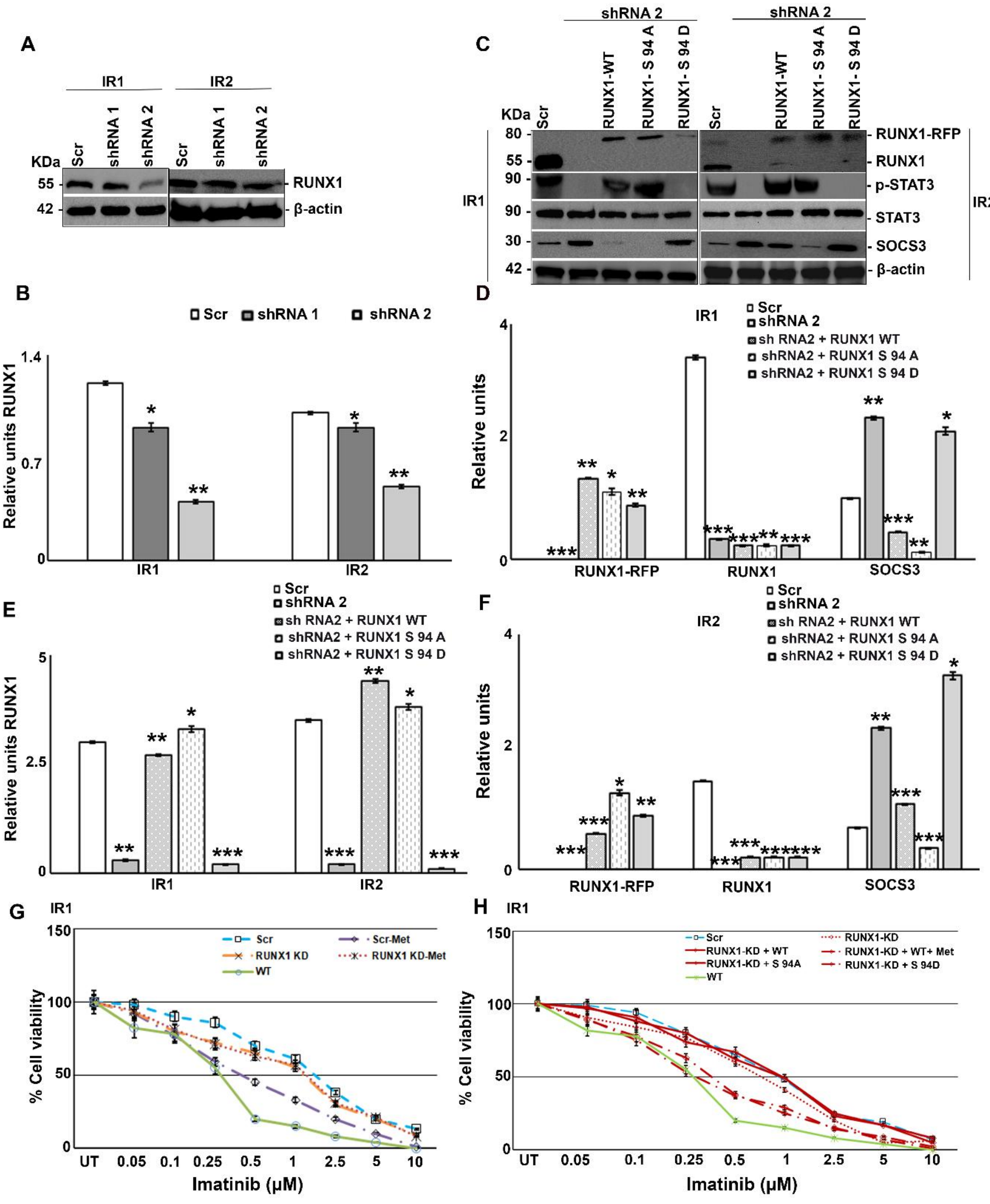

Supplementary figure 5

Supplementary fig. 5: A) Immunoblot analysis of K562 IR1 and IR2 cells, showing the efficiency of knockdown of RUNX1 by shRNAs. **B)** Quantification of immunoblot data. **C)**

Immunoblot analysis of K562 IR1 or IR2-RUNX1 KD (with shRNA 2), RUNX1 WT, RUNX1 S94A and RUNX1 S94D transduced cells showing levels of p-STAT3, STAT3, SOCS3, RUNX1 and RUNX1-RFP. **D-F)** Quantification of immunoblot data. Immunoblot analysis of K562 IR1 or IR2-RUNX1 KD (with shRNA 2), RUNX1 WT, RUNX1 S94A and RUNX1 S94D transduced cells showing levels of p-STAT3, STAT3, SOCS3, RUNX1 and RUNX1-RFP. **G)** Cell viability analysis of K562 IR1 cells transduced with either Scr or RUNX1 KD with or without metformin along with imatinib treatment, using WT as control. **H)** Cell viability analysis of K562 IR1-RUNX1 KD, RUNX1 WT, RUNX1 S94A and RUNX1 S94D transduced cells in response to imatinib treatment alone or along with metformin (0.25mM) for 72 hours with K562 WT as control, showing decreased cell viability in presence of metformin and in RUNX1 S94D cells which was reversed upon RUNX1 KD and in RUNX1 WT and RUNX1 S94A. N=3, Mean ± S.E.M. *$p<0.05$ versus control, $^{NS}p>0.05$.

Cont: control, Met: metformin, Ima: imatinib, WT: wild type, UT: untreated, Scr: scrambled, KD: knock down, NS: non-significant.

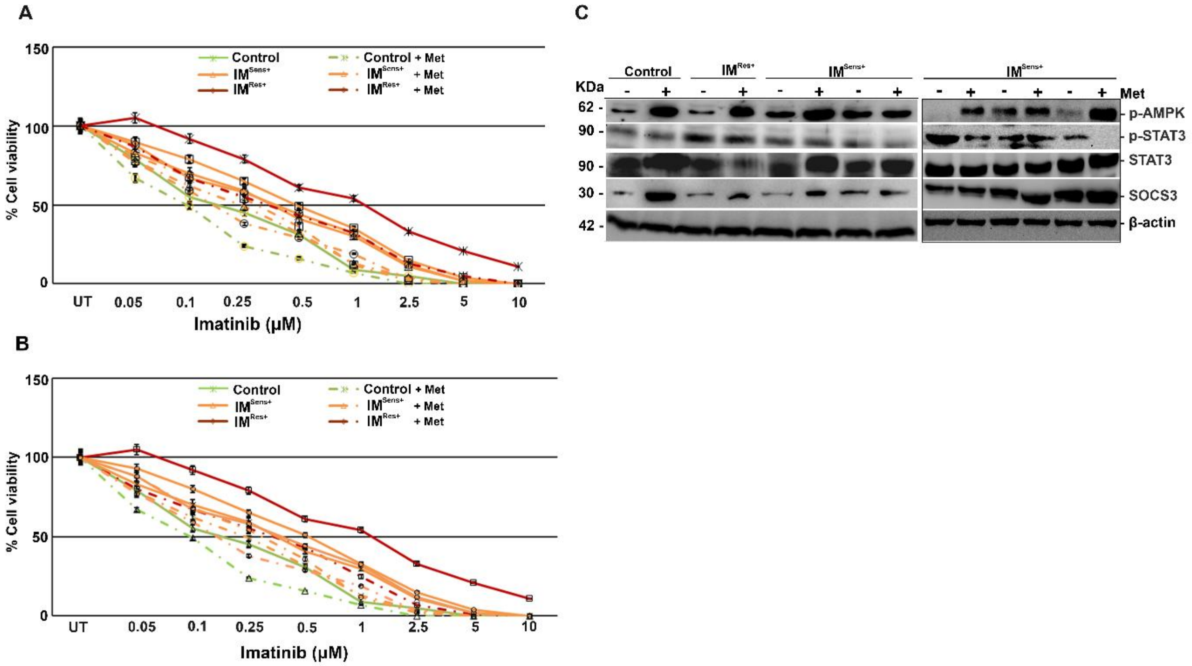

Supplementary figure 6

Supplementary fig. 6: A &B) Cell viability analysis of PBMCs post to treatment with imatinib alone or in combination with metformin (0.25mM) for 72 hours showing increased imatinib sensitivity. **C)** Immunoblot analysis of healthy and CML subject's PBMCs showing levels of p-STAT3, STAT3 and SOCS3 in response to metformin (10mM) treatment for 12 hours. N=3, Mean ± S.E.M.

Control: healthy subjects, IM^{Sens+}: Imatinib Sensitive CML subjects, IM $^{Res+}$: Imatinib resistant subjects, Met: metformin.

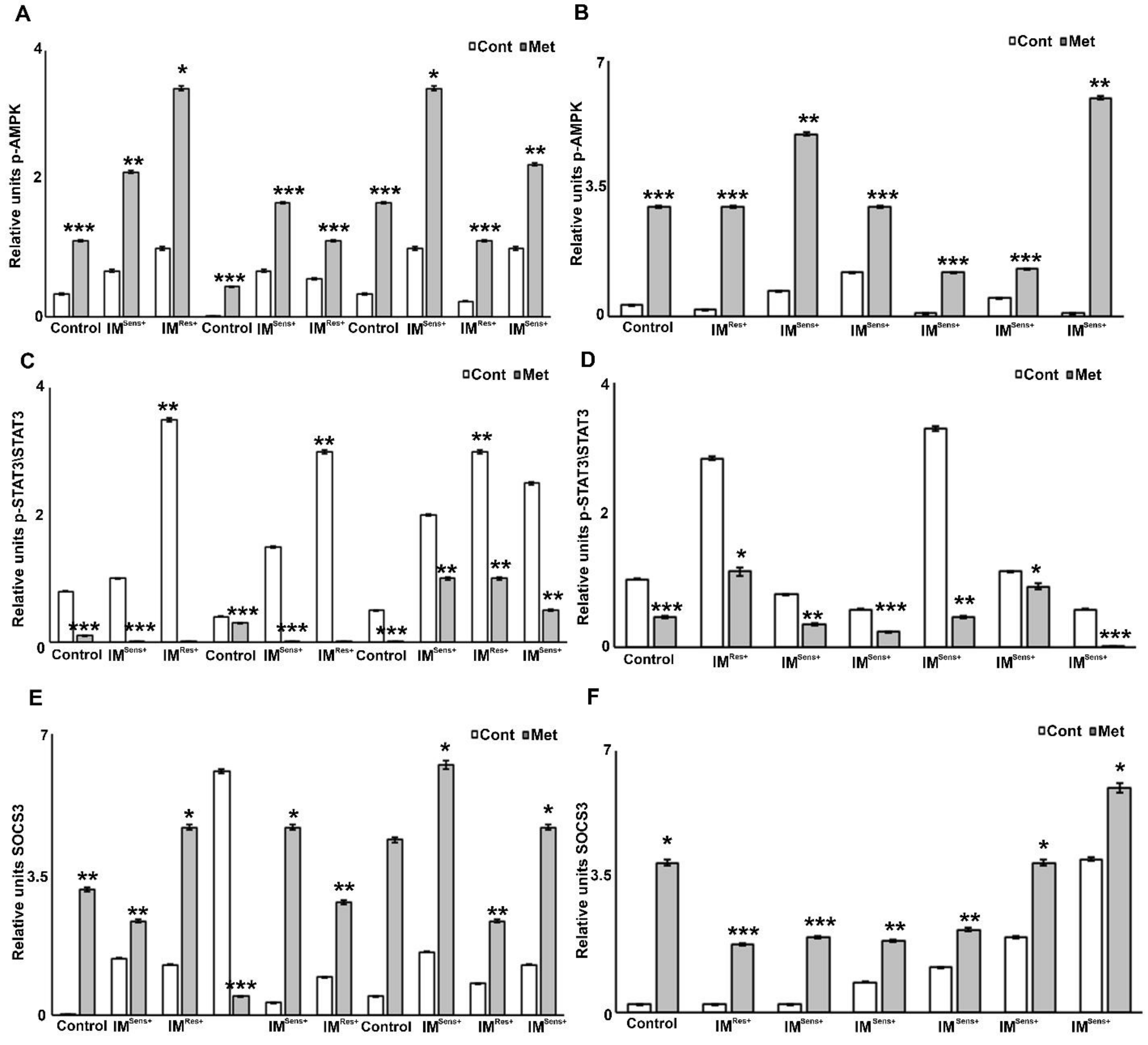

Supplementary figure 7

Supplementary fig. 7: Quantification of immunoblot data of PBMCs showing levels of **A &B)** p-AMPK, **C &D)** p-STAT3 and **E &F)** SOCS3 in response to metformin (10mM) treatment for 12 hours. N=3, Mean ± S.E.M. *$p<0.05$ versus control, **$p<0.005$ versus control, ***$p<0.0005$ versus control.

Cont: control, Met: metformin, control: healthy subjects, IM^{Sens+}: Imatinib Sensitive CML subjects, IM $^{Res+}$: Imatinib resistant subjects, NS: non-significant.
