# Supplementary material for: AMPK-induced novel phosphorylation of RUNX1 inhibits STAT3 activation and overcome imatinib resistance in chronic myelogenous leukemia (CML) subjects
Source: Cell Death Discov. 2023 Oct 30;9:401. doi: 10.1038/s41420-023-01700-x (PMC10616083; doi:10.1038/s41420-023-01700-x)
Supplement: Supplementary file 3 — Original Data File [file 41420_2023_1700_MOESM3_ESM.pdf]

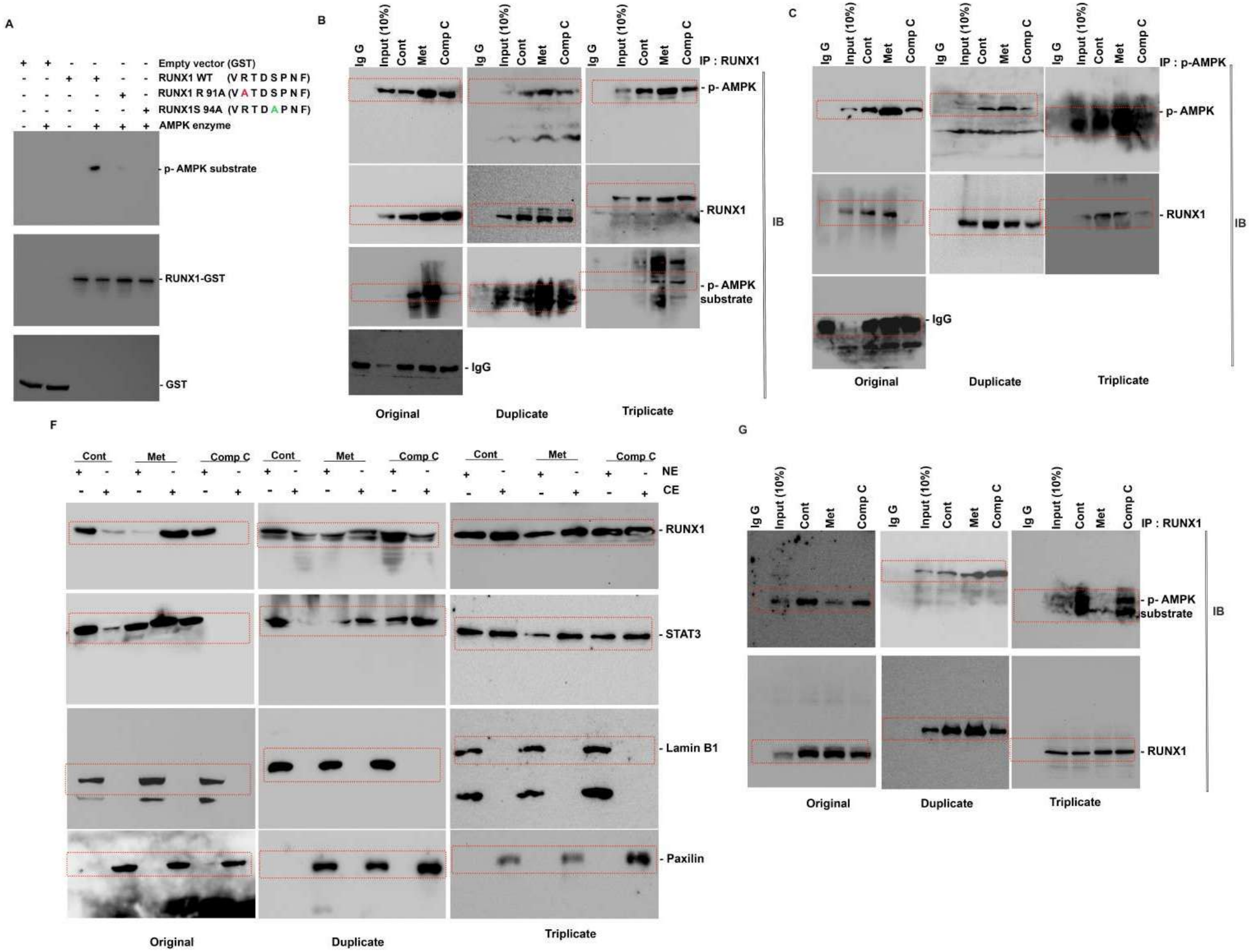
A
Empty vector (GST)
RUNX1 WT (V R T D S P N F)
RUNX1 R 91A (V A T D S P N F)
RUNX1S 94A (V R T D A P N F)
AMPK enzyme
p- AMPK substrate
RUNX1-GST
GST
B
Ig G
Input (10%)
Cont
Met
Comp C
IP : RUNX1
p- AMPK
RUNX1
p- AMPK substrate
IgG
IB
Original
Duplicate
Triplicate
C
IP : p-AMPK
F
NE
CE
STAT3
Lamin B1
Paxilin
G

Figure 2

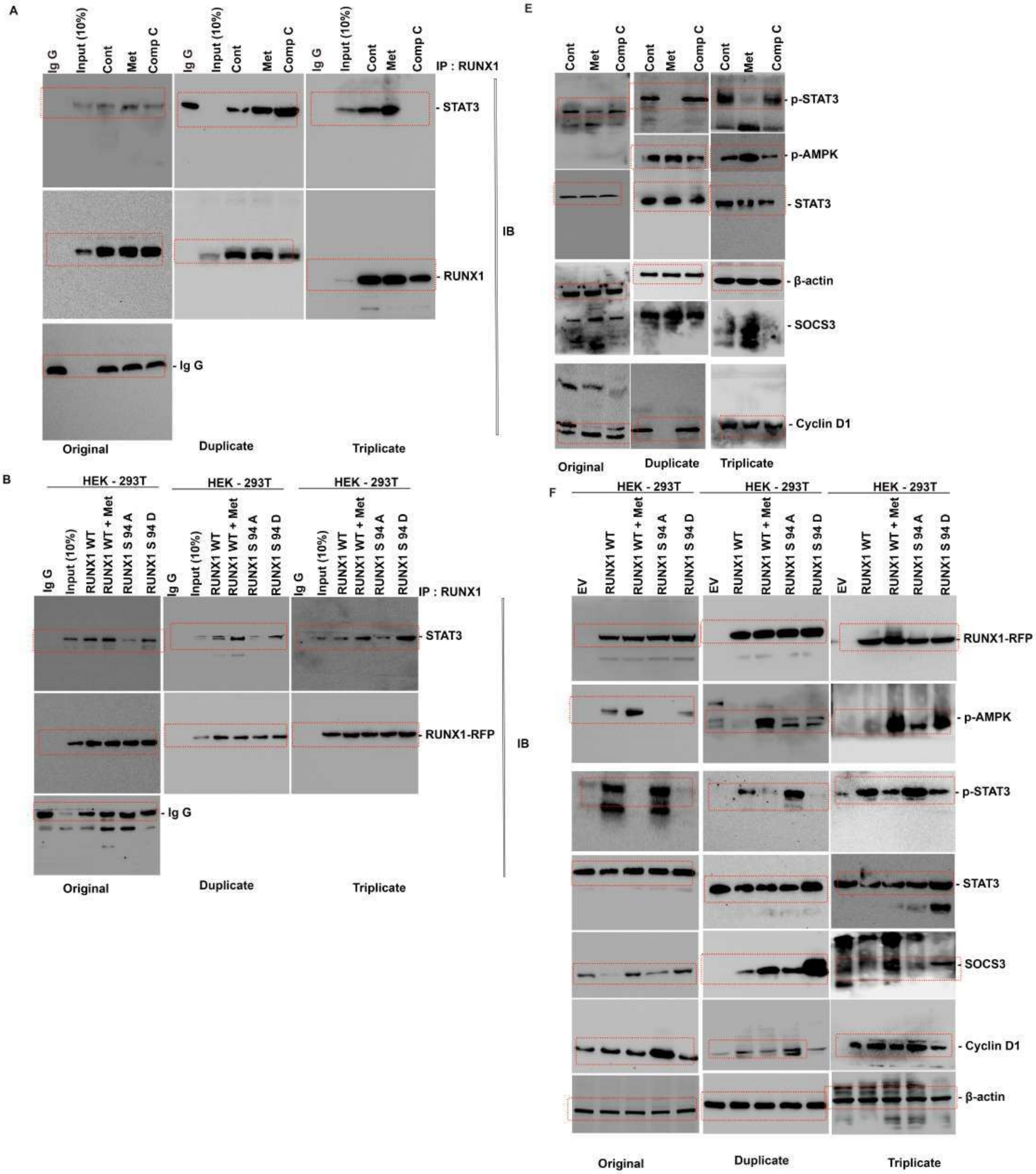

A
Ig G
Input (10%)
Cont
Met
Comp C
IP : RUNX1
STAT3
RUNX1
Ig G
IB
Original
Duplicate
Triplicate
B
HEK - 293T
Ig G
Input (10%)
RUNX1 WT
RUNX1 WT + Met
RUNX1 S 94 A
RUNX1 S 94 D
IP : RUNX1
STAT3
RUNX1-RFP
Ig G
IB
Original
Duplicate
Triplicate
E
Cont
Met
Comp C
p-STAT3
p-AMPK
STAT3
β-actin
SOCS3
Cyclin D1
Original
Duplicate
Triplicate
F
HEK - 293T
EV
RUNX1 WT
RUNX1 WT + Met
RUNX1 S 94 A
RUNX1 S 94 D
RUNX1-RFP
p-AMPK
p-STAT3
STAT3
SOCS3
Cyclin D1
β-actin
Original
Duplicate
Triplicate

Figure 3

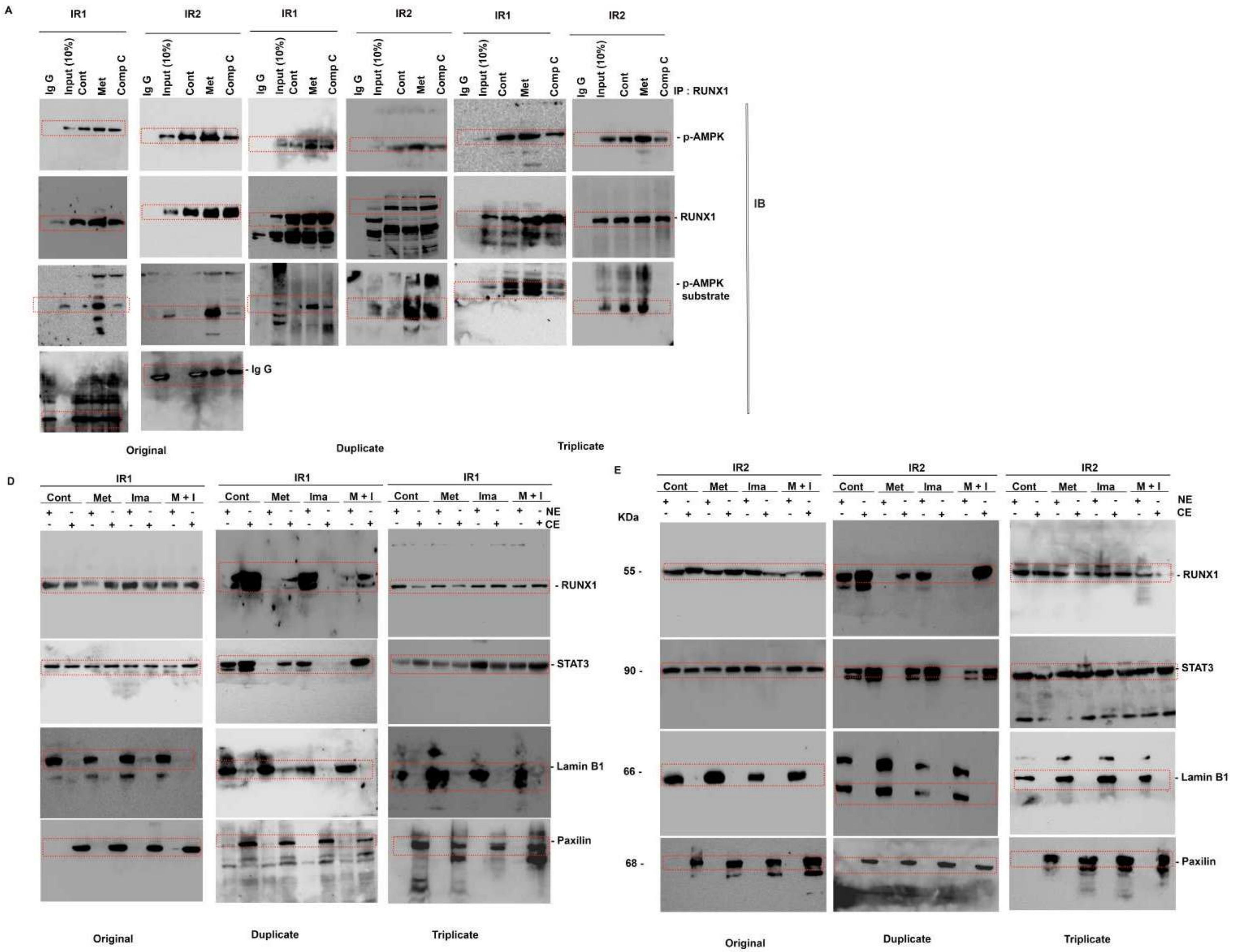
A
IR1
IR2
Ig G
Input (10%)
Cont
Met
Comp C
IP : RUNX1
- p-AMPK
- RUNX1
- p-AMPK substrate
- Ig G
IB
Original
Duplicate
Triplicate
D
E
Cont
Ima
M + I
NE
CE
KDa
55 -
90 -
66 -
68 -
- STAT3
- Lamin B1
- Paxilin

Figure 4

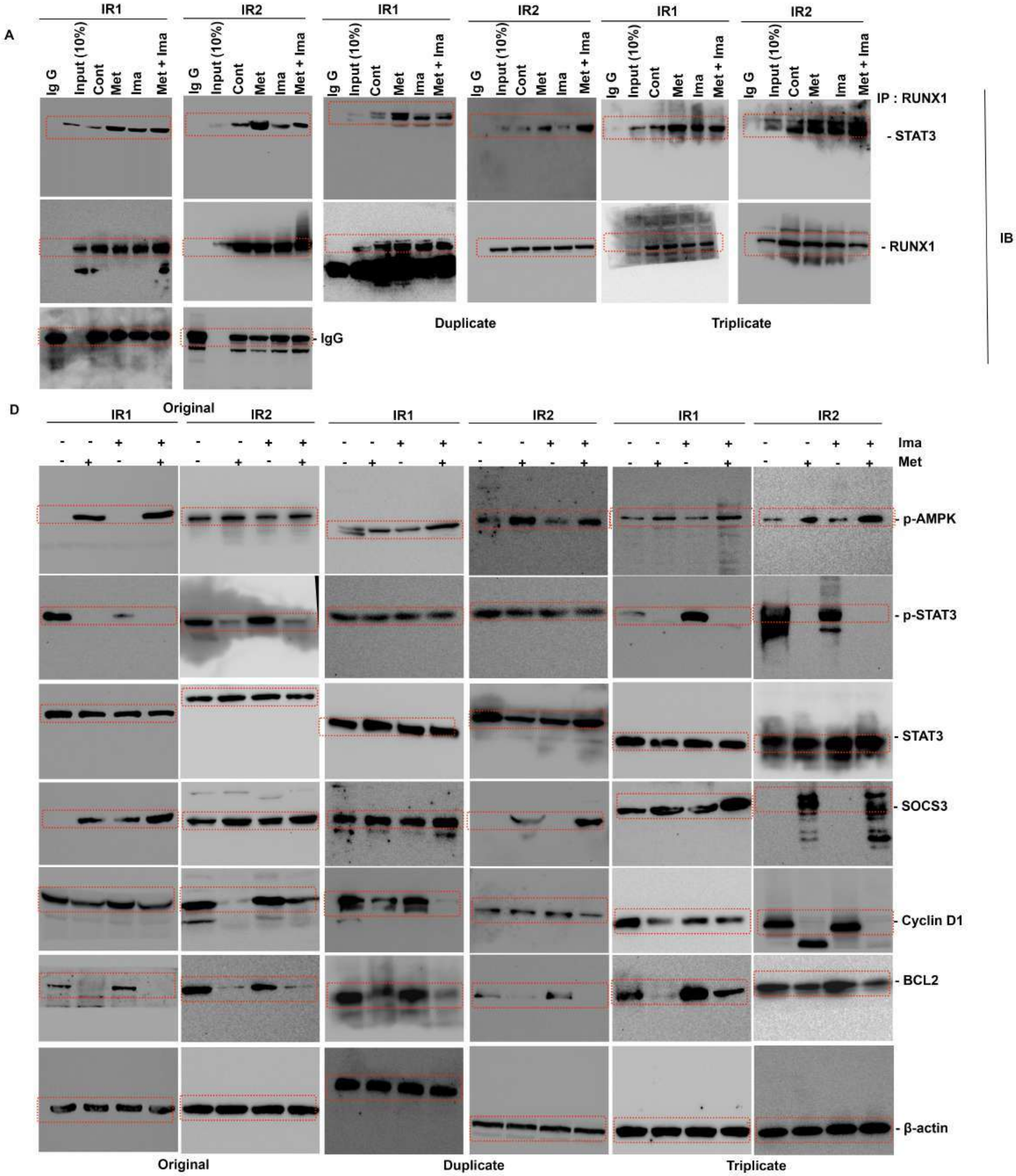
A
IR1
IR2
Ig G
Input (10%)
Cont
Met
Ima
Met + Ima
IP : RUNX1
- STAT3
- RUNX1
IB
- IgG
Original
Duplicate
Triplicate
D
Ima
Met
- p-AMPK
- p-STAT3
- STAT3
- SOCS3
- Cyclin D1
- BCL2
- β-actin

Figure 5

E

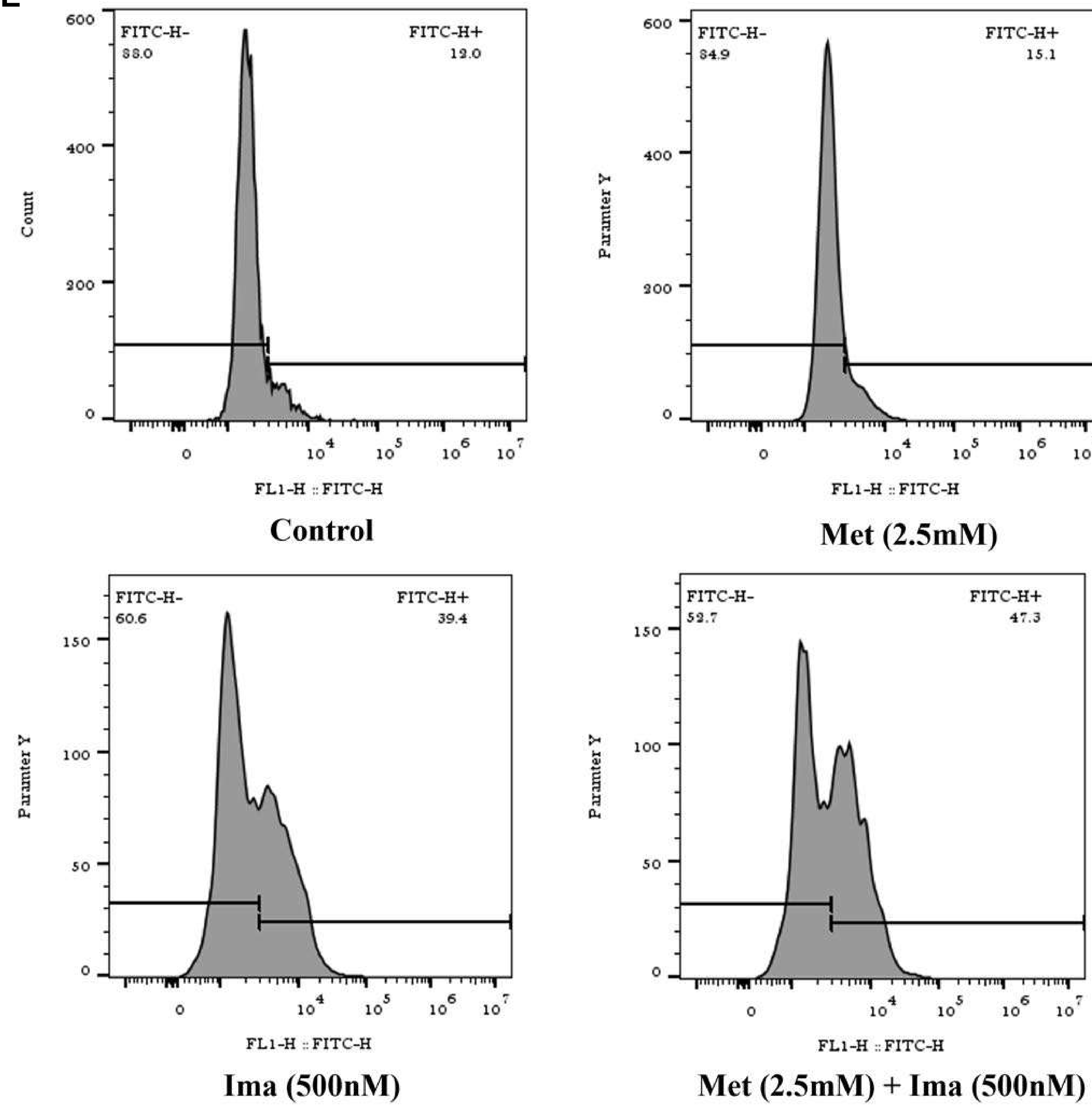
FITC-H-
88.0
FITC-H+
12.0
Count
FL1-H :: FITC-H
Control
FITC-H-
84.9
FITC-H+
15.1
Paramter Y
FL1-H :: FITC-H
Met (2.5mM)
FITC-H-
60.6
FITC-H+
39.4
Paramter Y
FL1-H :: FITC-H
Ima (500nM)
FITC-H-
52.7
FITC-H+
47.3
Paramter Y
FL1-H :: FITC-H
Met (2.5mM) + Ima (500nM)

Figure 5E

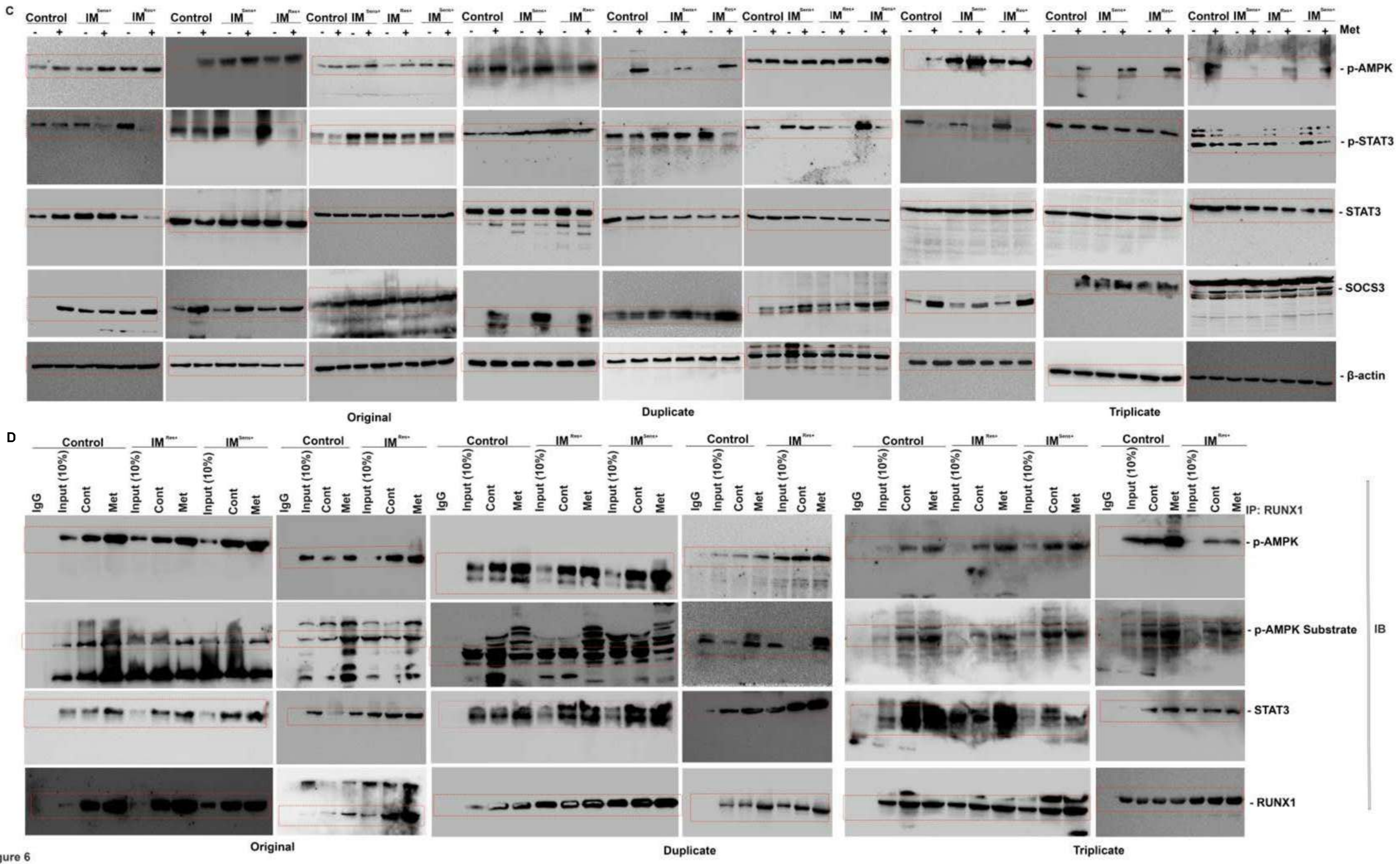
C
Control
IM Sens+
IM Res+
Met
- p-AMPK
- p-STAT3
- STAT3
- SOCS3
- β-actin
Original
Duplicate
Triplicate
D
IgG
Input (10%)
Cont
Met
IP: RUNX1
- p-AMPK
- p-AMPK Substrate
- STAT3
- RUNX1
IB

Figure 6

F

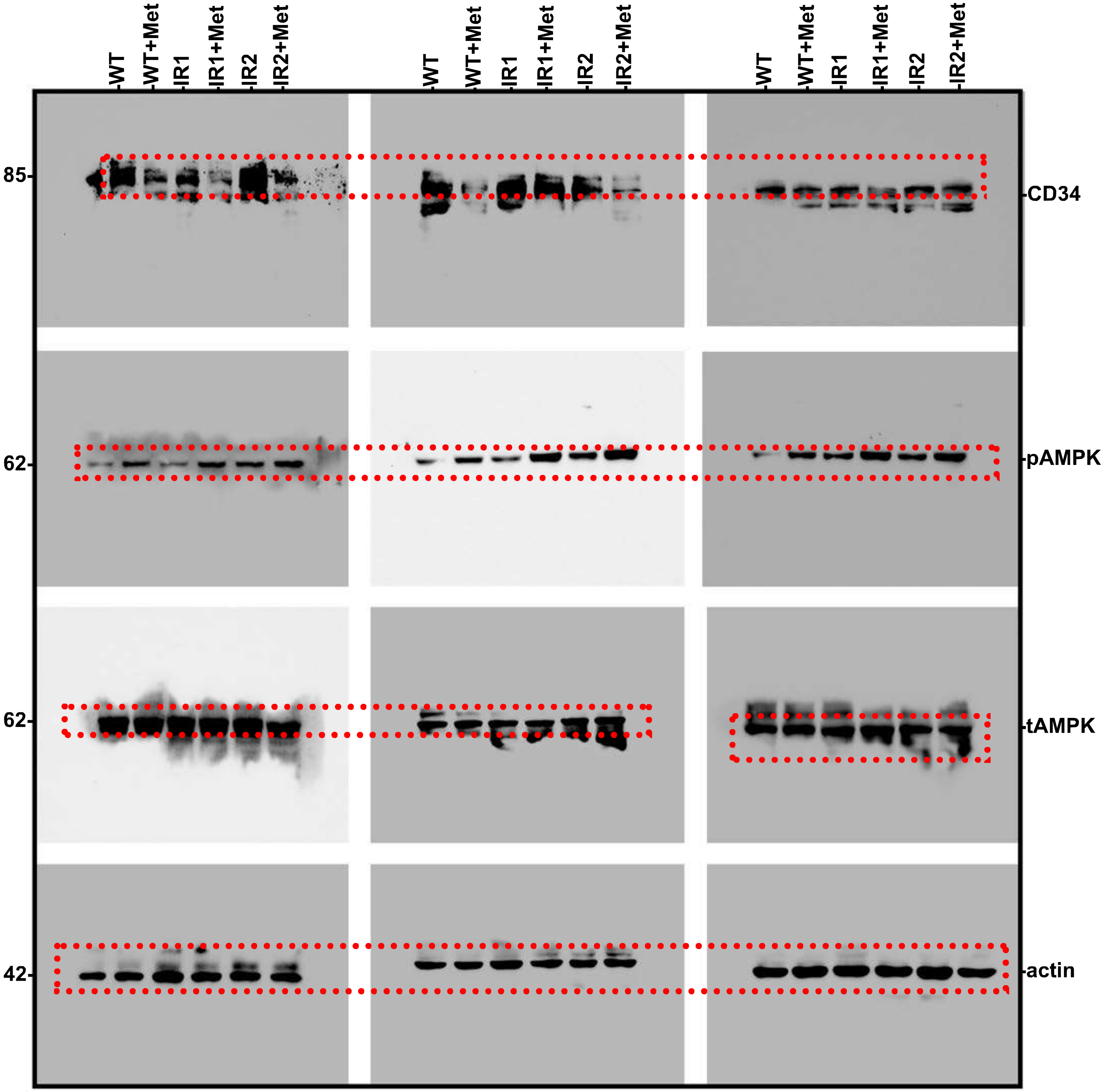
WT
WT+Met
IR1
IR1+Met
IR2
IR2+Met
WT
WT+Met
IR1
IR1+Met
IR2
IR2+Met
WT
WT+Met
IR1
IR1+Met
IR2
IR2+Met
85
62
62
42
CD34
pAMPK
tAMPK
actin

Figure 6

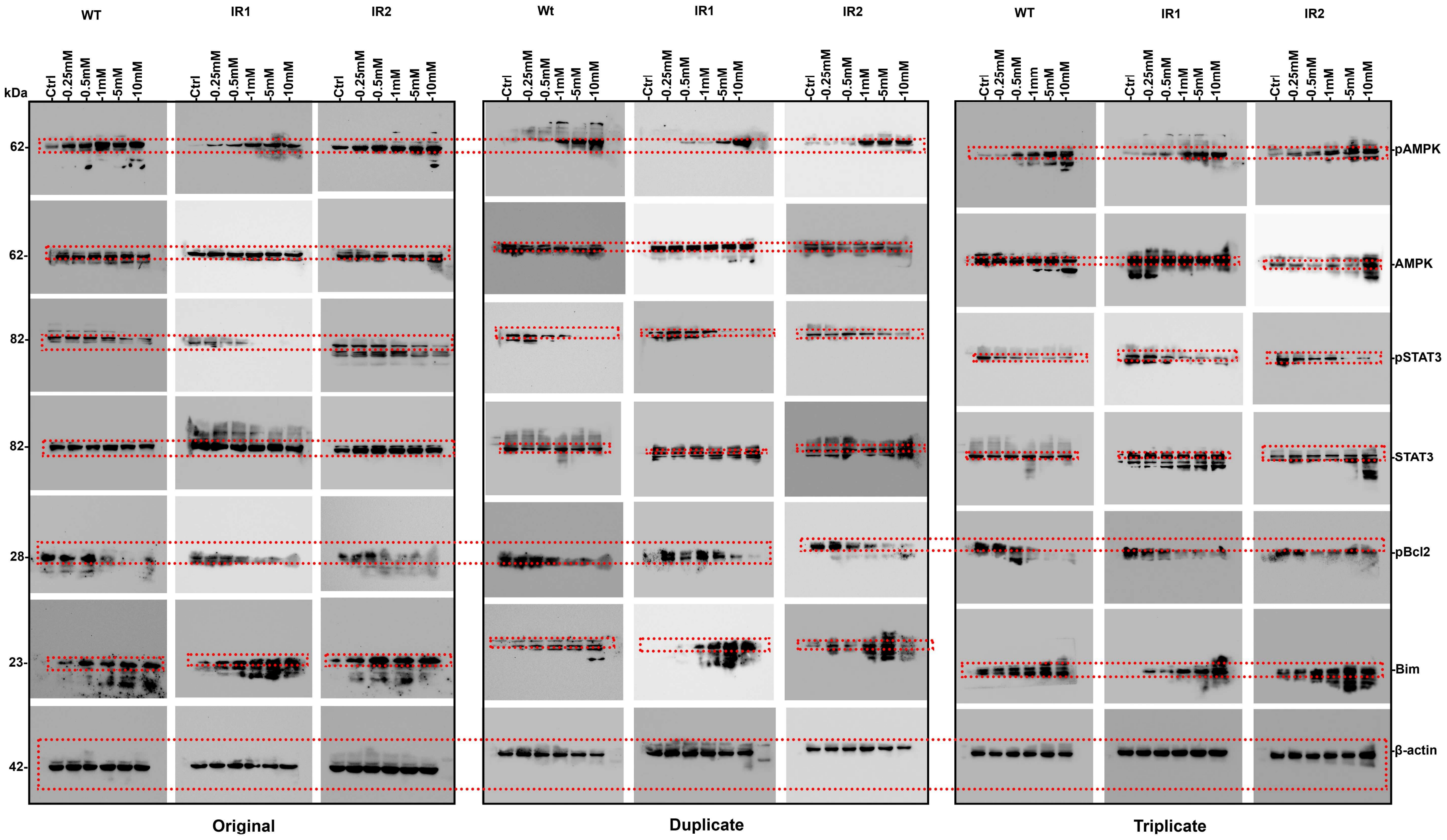

Supplementary figure 4

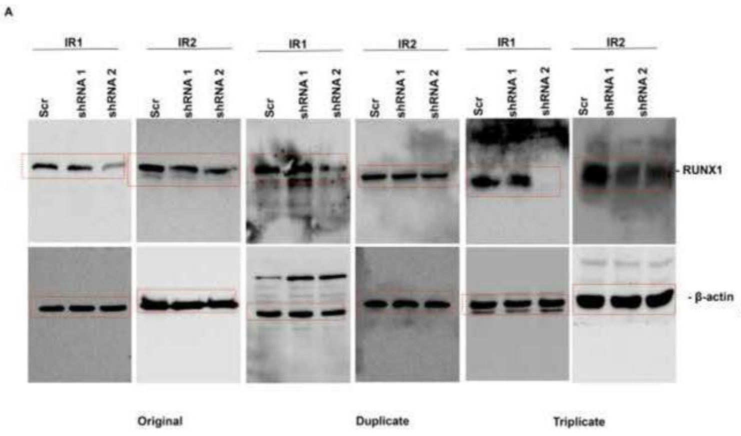

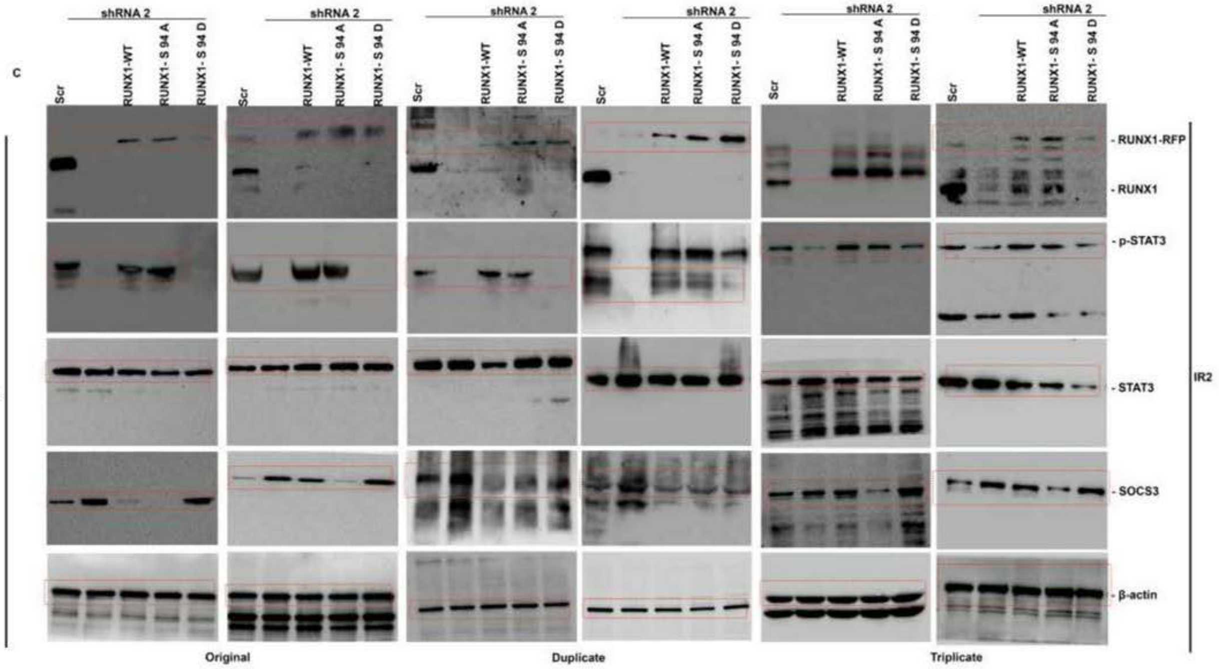

Supplementary Figure 5

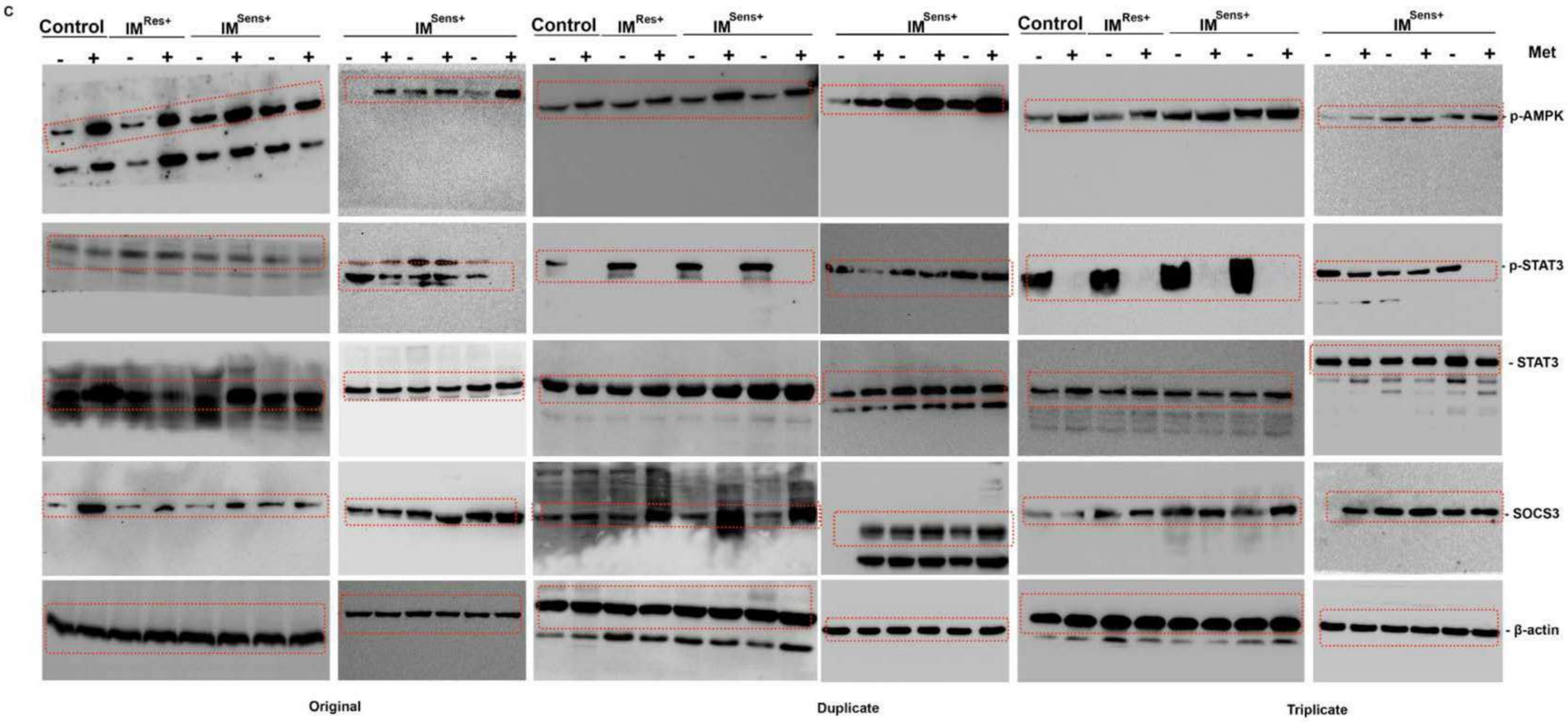

Supplementary Figure 6
